# Discovery of novel heart rate-associated loci using the Exome Chip

**DOI:** 10.1093/hmg/ddx113

**Published:** 2017-04-03

**Authors:** Marten E. van den Berg, Helen R. Warren, Claudia P. Cabrera, Niek Verweij, Borbala Mifsud, Jeffrey Haessler, Nathan A. Bihlmeyer, Yi-Ping Fu, Stefan Weiss, Henry J. Lin, Niels Grarup, Ruifang Li-Gao, Giorgio Pistis, Nabi Shah, Jennifer A. Brody, Martina Müller-Nurasyid, Honghuang Lin, Hao Mei, Albert V. Smith, Leo-Pekka Lyytikäinen, Leanne M. Hall, Jessica van Setten, Stella Trompet, Bram P. Prins, Aaron Isaacs, Farid Radmanesh, Jonathan Marten, Aiman Entwistle, Jan A. Kors, Claudia T. Silva, Alvaro Alonso, Joshua C. Bis, Rudolf de Boer, Hugoline G. de Haan, Renée de Mutsert, George Dedoussis, Anna F. Dominiczak, Alex S. F. Doney, Patrick T. Ellinor, Ruben N. Eppinga, Stephan B. Felix, Xiuqing Guo, Yanick Hagemeijer, Torben Hansen, Tamara B. Harris, Susan R. Heckbert, Paul L. Huang, Shih-Jen Hwang, Mika Kähönen, Jørgen K. Kanters, Ivana Kolcic, Lenore J. Launer, Man Li, Jie Yao, Allan Linneberg, Simin Liu, Peter W. Macfarlane, Massimo Mangino, Andrew D. Morris, Antonella Mulas, Alison D. Murray, Christopher P. Nelson, Marco Orrú, Sandosh Padmanabhan, Annette Peters, David J. Porteous, Neil Poulter, Bruce M. Psaty, Lihong Qi, Olli T. Raitakari, Fernando Rivadeneira, Carolina Roselli, Igor Rudan, Naveed Sattar, Peter Sever, Moritz F. Sinner, Elsayed Z. Soliman, Timothy D. Spector, Alice V. Stanton, Kathleen E. Stirrups, Kent D. Taylor, Martin D. Tobin, André Uitterlinden, Ilonca Vaartjes, Arno W. Hoes, Peter van der Meer, Uwe Völker, Melanie Waldenberger, Zhijun Xie, Magdalena Zoledziewska, Andrew Tinker, Ozren Polasek, Jonathan Rosand, Yalda Jamshidi, Cornelia M. van Duijn, Eleftheria Zeggini, J. Wouter Jukema, Folkert W. Asselbergs, Nilesh J. Samani, Terho Lehtimäki, Vilmundur Gudnason, James Wilson, Steven A. Lubitz, Stefan Kääb, Nona Sotoodehnia, Mark J. Caulfield, Colin N. A. Palmer, Serena Sanna, Dennis O. Mook-Kanamori, Panos Deloukas, Oluf Pedersen, Jerome I. Rotter, Marcus Dörr, Chris J. O'Donnell, Caroline Hayward, Dan E. Arking, Charles Kooperberg, Pim van der Harst, Mark Eijgelsheim, Bruno H. Stricker, Patricia B. Munroe

**Affiliations:** 1 Department of Medical Informatics Erasmus MC - University Medical Center Rotterdam, P.O. Box 2040, 3000CA, Rotterdam, the Netherlands,; 2 Clinical Pharmacology, William Harvey Research Institute, Queen Mary University of London, London, EC1M 6BQ, UK,; 3 NIHR Barts Cardiovascular Biomedical Research Unit, Queen Mary University of London, London, EC1M 6BQ, UK,; 4 University Medical Center Groningen, University of Groningen, Department of Cardiology, the Netherlands,; 5 Division of Public Health Sciences, Fred Hutchinson Cancer Research Center, Seattle, WA 98109, USA,; 6 Predoctoral Training Program in Human Genetics, McKusick-Nathans Institute of Genetic Medicine, Johns Hopkins University School of Medicine, Baltimore, MD, USA, 21205,; 7 Division of Cardiovascular Sciences, National Heart, Lung, and Blood Institute, National Institutes of Health, Bethesda, MD, USA ,; 8 Interfaculty Institute for Genetics and Functional Genomics; University Medicine and Ernst-Moritz-Arndt-University Greifswald; Greifswald, 17475, Germany,; 9 DZHK (German Centre for Cardiovascular Research); partner site Greifswald; Greifswald, 17475, Germany,; 10 The Institute for Translational Genomics and Population Sciences, Department of Pediatrics, Los Angeles Biomedical Research Institute at Harbor-UCLA Medical Center, 1124 W. Carson Street, Torrance, CA 90502, USA,; 11 Division of Medical Genetics, Department of Pediatrics, Harbor-UCLA Medical Center, Torrance, CA, USA,; 12 The Novo Nordisk Foundation Center for Basic Metabolic Research, Faculty of Health and Medical Sciences, University of Copenhagen, Copenhagen, Denmark,; 13 Department of Clinical Epidemiology, Leiden University Medical Center, Leiden, the Netherlands,; 14 Istituto di Ricerca Genetica e Biomedica (IRGB), CNR, Monserrato, Italy ,; 15 Center for Statistical Genetics, University of Michigan, Ann Arbor, MI, USA,; 16 Division of Molecular and Clinical Medicine, School of Medicine, University of Dundee, DD1 9SY, UK,; 17 Department of Pharmacy, COMSATS Institute of Information Technology, Abbottabad, 22060, Pakistan,; 18 Cardiovascular Health Research Unit, Department of Medicine, University of Washington, 1730 Minor Ave, Suite 1360, Seattle, WA 98101, USA,; 19 Institute of Genetic Epidemiology, Helmholtz Zentrum München - German Research Center for Environmental Health, Neuherberg, Germany,; 20 DZHK (German Centre for Cardiovascular Research), partner site Munich Heart Alliance, Munich, Germany,; 21 Department of Medicine I, University Hospital Munich, Ludwig-Maximilians-Universität, Munich, Germany,; 22 Section of Computational Biomedicine, Department of Medicine, Boston University School of Medicine, Boston, MA,; 23 Department of Data Science, University of Mississippi Medical Center, Jackson, MI, USA,; 24 Icelandic Heart Association, 201 Kopavogur, Iceland,; 25 Faculty of Medicine, University of Iceland, 101 Reykjavik, Iceland,; 26 Department of Clinical Chemistry, Fimlab Laboratories and University of Tampere School of Medicine, Arvo, D339, P.O. Box 100, FI-33014 Tampere, Finland,; 27 Department of Cardiovascular Sciences, University of Leicester, Cardiovascular Research Centre, Glenfield Hospital, Leicester, LE3 9QP, UK,; 28 NIHR Leicester Cardiovascular Biomedical Research Unit, Glenfield Hospital, Leicester LE3 9QP, UK,; 29 Department of Cardiology, Division Heart & Lungs, University Medical Center Utrecht, Utrecht, the Netherlands,; 30 Department of Cardiology, Leiden University Medical Center, 2300 RC, Leiden, the Netherlands,; 31 Department of Gerontology and Geriatrics, Leiden university Medical Center, Leiden, the Netherlands,; 32 Department of Human Genetics, Wellcome Trust Sanger Institute, Hinxton, United Kingdom, CB10 1SA,; 33 Cardiogenetics Lab, Genetics and Molecular Cell Sciences Research Centre, Cardiovascular and Cell Sciences Institute, St George’s, University of London, Cranmer Terrace, London, SW17 0RE, UK,; 34 CARIM School for Cardiovascular Diseases, Maastricht Centre for Systems Biology (MaCSBio), Dept. of Biochemistry, Maastricht University, Universiteitssingel 60, 6229 ER Maastricht, NL,; 35 Center for Human Genetic Research, Massachusetts General Hospital, Boston, MA 02114,; 36 Program in Medical and Population Genetics, Broad Institute, Cambridge, MA 02142,; 37 MRC Human Genetics Unit, MRC Institute of Genetics and Molecular Medicine, University of Edinburgh, Western General Hospital, Crewe Road South, Edinburgh, EH4 2XU, UK,; 38 Genetic Epidemiology Unit, Dept. of Epidemiology, Erasmus University Medical Center, PO Box 2040, 3000 CA Rotterdam, NL,; 39 Doctoral Program in Biomedical Sciences, Universidad del Rosario, Bogotá, Colombia,; 40 GENIUROS Group, Genetics and Genomics Research Center CIGGUR, School of Medicine and Health Sciences, Universidad del Rosario, Bogotá, Colombia,; 41 Department of Epidemiology, Rollins School of Public Health, Emory University, Atlanta, GA, 30322,; 42 Department of Nutrition and Dietetics, School of Health Science and Education, Harokopio University, Athens 17671, Greece,; 43 Institute of Cardiovascular and Medical Sciences, College of Medical, Veterinary and Life Sciences, University of Glasgow, Glasgow, UK,; 44 Cardiovascular Research Center, Massachusetts General Hospital, Charlestown, MA, USA,; 45 Department of Internal Medicine B - Cardiology, Pneumology, Infectious Diseases, Intensive Care Medicine; University Medicine Greifswald; Greifswald, 17475, Germany & DZHK (German Centre for Cardiovascular Research); partner site Greifswald; Greifswald, 17475, Germany,; 46 Laboratory of Epidemiology and Population Sciences, National Institute on Aging, Intramural Research Program, National Institutes of Health, Bethesda, Maryland, 20892, USA,; 47 Cardiovascular Health Research Unit and Department of Epidemiology, University of Washington, 1730 Minor Ave, Suite 1360, Seattle, WA 98101, USA,; 48 Group Health Research Institute, Group Health Cooperative, 1730 Minor Ave, Suite 1600, Seattle, WA, USA,; 49 Population Sciences Branch, Division of Intramural Research, NHLBI, NIH, Bethesda MD, USA,; 50 Department of Clinical Physiology, Tampere University Hospital and University of Tampere School of Medicine, Finn-Medi 1, 3th floor, P.O. Box 2000, FI-33521 Tampere, Finland,; 51 Laboratory of Experimental Cardiology, University of Copenhagen, Copenhagen, Denmark,; 52 Faculty of Medicine, University of Split, Split, Croatia,; 53 Division of Nephrology & Hypertension, Internal Medicine, School of Medicine, University of Utah, Salt Lake City, UT 84109, USA,; 54 Research Centre for Prevention and Health, Capital Region of Denmark, Copenhagen, Denmark,; 55 Department of Clinical Experimental Research, Rigshospitalet, Glostrup, Denmark,; 56 Department of Clinical Medicine, Faculty of Health and Medical Sciences, University of Copenhagen, Copenhagen, Denmark,; 57 Brown University School of Public Health, Providence, Rhode Island 02912, USA,; 58 Institute of Health and Wellbeing, University of Glasgow, Glasgow, UK,; 59 Department of Twin Research and Genetic Epidemiology, King's College London, London, UK,; 60 NIHR Biomedical Research Centre at Guy’s and St Thomas’ Foundation Trust, London SE1 9RT, UK,; 61 Usher Institute of Population Health Sciences and Informatics, University of Edinburgh, Edinburgh, EH8 9AG, UK,; 62 Aberdeen Biomedical Imaging Centre, Lilian Sutton Building, University of Aberdeen, Foresterhill, Aberdeen AB25 2ZD, UK,; 63 Unita Operativa Complessa di Cardiologia, Presidio Ospedaliero Oncologico Armando Businco Cagliari , Azienda Ospedaliera Brotzu Cagliari, Caglliari, Italy,; 64 Institute of Cardiovascular and Medical Sciences, University of Glasgow, BHF GCRC, Glasgow G12 8TA, UK,; 65 Institute of Epidemiology II, Helmholtz Zentrum München - German Research Center for Environmental Health, Neuherberg, Germany,; 66 German Center for Diabetes Research, Neuherberg, Germany,; 67 Centre for Genomic & Experimental Medicine, Institute of Genetics & Molecular Medicine, University of Edinburgh, Western General Hospital, Crewe Road South, Edinburgh EH4 2XU, UK,; 68 School of Public Health, Imperial College London, W2 1PG, UK,; 69 Cardiovascular Health Research Unit, Department of Health Services, University of Washington, 1730 Minor Ave, Suite 1360, Seattle, WA 98101, USA,; 70 Group Health Research Institute, Group Health Cooperative, Seattle, WA, USA,; 71 University of California Davis, One Shields Ave Ms1c 145, Davis, CA 95616 USA,; 72 Department of Clinical Physiology and Nuclear Medicine, Turku University Hospital, and Research Centre of Applied and Preventive Cardiovascular Medicine, University of Turku, P.O. Box 52, FI-20521 Turku, Finland,; 73 Human Genomics Facility Erasmus MC - University Medical Center Rotterdam, P.O. Box 2040, 3000CA, Rotterdam, the Netherlands,; 74 Program in Medical and Population Genetics, Broad Institute of MIT and Harvard, Cambridge, MA, USA,; 75 National Heart and Lung Institute, Imperial College London, W2 1PG, UK,; 76 Epidemiological Cardiology Research Center (EPICARE), Wake Forest School of Medicine, Winston-Salem, NC 27157, USA,; 77 Molecular and Cellular Therapeutics, Royal College of Surgeons in Ireland, Dublin 2, Ireland,; 78 Department of Haematology, University of Cambridge, Cambridge, UK,; 79 Institute for Translational Genomics and Population Sciences, Los Angeles BioMedical Research Institute at Harbor-UCLA Medical Center, Torrance, CA, USA,; 80 Division of Genomic Outcomes, Department of Pediatrics, Harbor-UCLA Medical Center, Torrance, CA, USA,; 81 Departments of Pediatrics, Medicine, and Human Genetics, UCLA, Los Angeles, CA, USA,; 82 Department of Health Sciences, University of Leicester, Leicester LE1 7RH, UK,; 83 Human Genotyping Facility Erasmus MC - University Medical Center Rotterdam, P.O. Box 2040, 3000CA, Rotterdam, the Netherlands,; 84 Julius Center for Health Sciences and Primary Care, University Medical Center, PO Box 85500, 3508 GA Utrecht, the Netherlands,; 85 Research Unit of Molecular Epidemiology, Helmholtz Zentrum München - German Research Center for Environmental Health, Neuherberg, Germany,; 86 Durrer Center for Cardiogenetic Research, ICIN-Netherlands Heart Institute, Utrecht, the Netherlands,; 87 Institute of Cardiovascular Science, Faculty of Population Health Sciences, University College London, London, UK,; 88 Department of Clinical Chemistry, Fimlab Laboratories and University of Tampere School of Medicine, Arvo, D338, P.O. Box 100, FI-33014 Tampere, Finland,; 89 Physiology & Biophysics, University of Mississippi Medical Center, Jackson, MI, USA,; 90 Cardiovascular Health Research Unit, Division of Cardiology, Departments of Medicine and Epidemiology, University of Washington, 1730 Minor Ave, Suite 1360, Seattle, WA 98101, USA,; 91 Department of Public Health and Primary Care, Leiden University Medical Center, Leiden, the Netherlands,; 92 The Institute for Translational Genomics and Population Sciences, Departments of Pediatrics and Medicine, Los Angeles Biomedical Research Institute at Harbor-UCLA Medical Center, 1124 W. Carson Street, Torrance, CA 90502, USA,; 93 Boston Veteran’s Administration Healthcare, Boston MA, USA,; 94 McKusick-Nathans Institute of Genetic Medicine, Johns Hopkins University School of Medicine, Baltimore, MD, USA, 21205 and; 95 Department of Epidemiology Erasmus MC - University Medical Center Rotterdam, P.O. Box 2040, 3000CA, Rotterdam, the Netherlands

## Abstract

Resting heart rate is a heritable trait, and an increase in heart rate is associated with increased mortality risk. Genome-wide association study analyses have found loci associated with resting heart rate, at the time of our study these loci explained 0.9% of the variation. This study aims to discover new genetic loci associated with heart rate from Exome Chip meta-analyses.

Heart rate was measured from either elecrtrocardiograms or pulse recordings. We meta-analysed heart rate association results from 104 452 European-ancestry individuals from 30 cohorts, genotyped using the Exome Chip. Twenty-four variants were selected for follow-up in an independent dataset (UK Biobank, *N* = 134 251). Conditional and gene-based testing was undertaken, and variants were investigated with bioinformatics methods.

We discovered five novel heart rate loci, and one new independent low-frequency non-synonymous variant in an established heart rate locus (*KIAA1755*). Lead variants in four of the novel loci are non-synonymous variants in the genes *C10orf71, DALDR3, TESK2* and *SEC31B*. The variant at *SEC31B* is significantly associated with *SEC31B* expression in heart and tibial nerve tissue. Further candidate genes were detected from long-range regulatory chromatin interactions in heart tissue (*SCD*, *SLF2* and *MAPK8*). We observed significant enrichment in DNase I hypersensitive sites in fetal heart and lung. Moreover, enrichment was seen for the first time in human neuronal progenitor cells (derived from embryonic stem cells) and fetal muscle samples by including our novel variants.

Our findings advance the knowledge of the genetic architecture of heart rate, and indicate new candidate genes for follow-up functional studies.

## Introduction

Increased resting heart rate (HR) is a known risk factor for cardiovascular morbidity and mortality (1–3), including stroke (4) and sudden cardiac death (5,6). Heart rate increased by 20 beats per minute (BPM) is associated with 30-50% higher mortality and appears to be independent of confounder factors (7). High HR increases myocardial oxygen consumption yet lessens oxygen delivery to myocardial tissue. It also increases arterial stiffness and risk of plaque rupture (8). Although HR can be influenced by many non-genetic factors (e.g. exercise, smoking and cardiovascular drugs), the heritability of resting HR is estimated to be 26–32% from family studies (9,10), and 55–63% from twin studies (11).

Several meta-analyses of genome-wide association studies (GWASs) have been undertaken to detect genetic determinants of HR (12–16). There were 21 HR loci previously reported at the time of our study by den Hoed *et al.* (12) in a GWAS analysis of 180 000 individuals, predominantly of European ancestry. The study implicated 20 candidate genes from follow-up functional studies in *Danio rerio* and *Drosophila melanogaster* models. Smaller GWAS analyses have also been performed in Icelandic and Norwegian populations (15), African Americans (13) and genetically isolated European populations (16). The variants discovered by GWAS are common, and are mostly in introns or intergenic regions. Together the previous loci from GWAS at the time of our study only explain a small percentage [0.9% of the variability in HR (12,17)].

To increase our knowledge of genetic determinants influencing HR and discover novel loci, especially rare or low frequency coding variants with larger effects, we meta-analysed data from 104 452 individuals of European-ancestry using the Exome Chip, from cohorts that participated in the Cohorts for Heart & Aging Research in Genomic Epidemiology (CHARGE) EKG consortium. The Exome Chip permits a cost-efficient analysis of coding variants derived from sequencing of >12 000 individuals and includes many rare and low-frequency variants (18). We performed a validation experiment using independent replication samples from UK Biobank data, and bioinformatics investigations to gain an understanding of the new HR loci.

## Results

### Single-nucleotide variant analysis in individuals of European-ancestry

**Figure 1:**
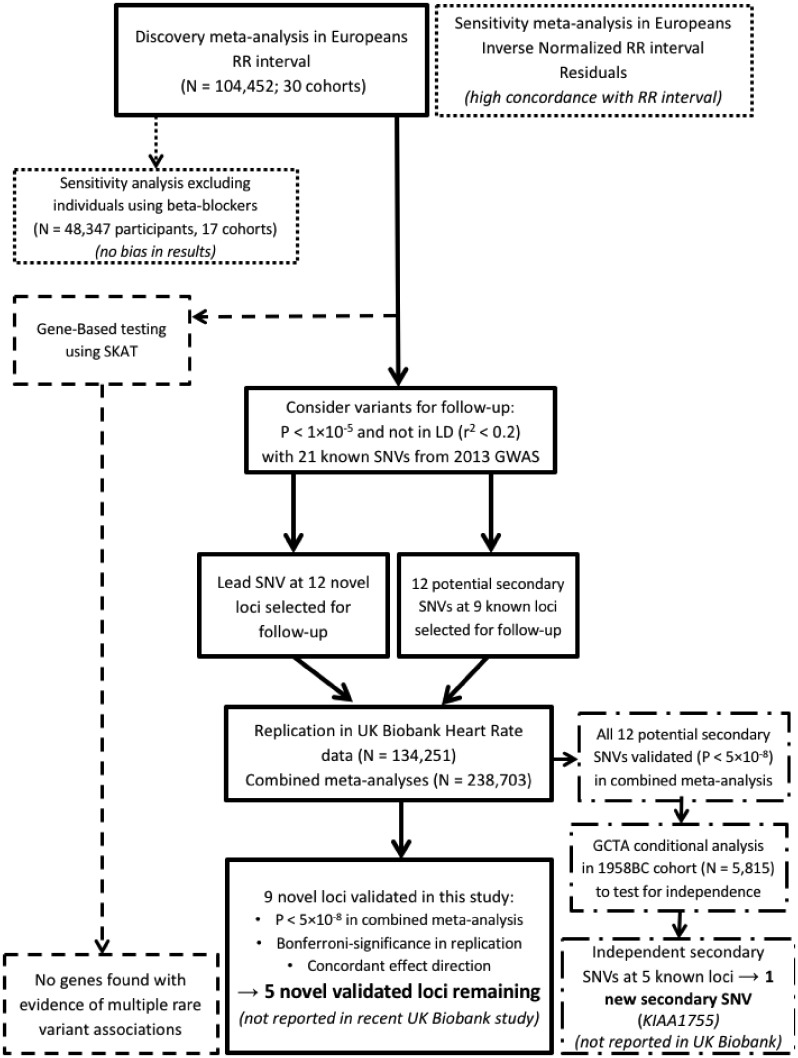
Schematic flow diagram of the study design. *N*, sample size; SKAT, SNV-set Kernel Association Test; *P*, *P*-value; LD, linkage disequilibrium; SNV, single nucleotide variant; GCTA, Genome-wide Complex Traits Analysis software; 1958BC, 1958 Birth Cohort; UKB, UK Biobank.

In the discovery phase, association results of 235 677 single-nucleotide variants (SNVs) from 104 452 individuals were meta-analysed using a fixed-effects model (Supplementary Material, Fig. S1). Two analyses were performed. The first used RR-intervals (RR in milliseconds= 60 000/HR, in beats per minute, according to the inverse relationship between HR and RR). The second used the inverse-normalized residuals of the linear regression RR-interval adjusted for age + sex + body mass index (BMI) as covariates (denoted as RR-INVN). An overview of the study design is provided in Figure 1.

We observed a high correlation of effect sizes and *P*-values between the RR-interval and RR-INVN meta-analyses (*r*^2^ = 0.99 and 0.98, respectively; Supplementary Material, Fig. S2). Furthermore, the RR-interval was near-normally distributed, so inverse normalization was deemed unnecessary (Supplementary Material, Fig. S3).

Beta-blockers are clinically known to lower HR, therefore the phenotype measurements of beta-blocker users may be under-estimated, and hence the inclusion of beta-blocker users in our analysis may potentially bias our analysis results. We therefore performed a sensitivity analysis by also meta-analysing a subgroup of cohorts that provided beta-blocker data (*N* = 48 347; 17 cohorts). Results including or excluding beta-blocker users were highly correlated (*r*^2^ of the betas = 0.97; *r*^2^ of the *P*-values = 0.74; Supplementary Material, Fig. S4), suggesting there is little or no bias from including beta-blocker users in the analysis. Therefore we report the meta-analysis results from the full dataset for the RR-interval, to maximize sample size and power.

### Replication and meta-analysis with the UK Biobank dataset

To identify novel associated loci, we selected 12 variants with *P* < 1 × 10^−5^ that mapped outside the 21 HR loci reported in the previous GWAS (12) for follow-up in an independent dataset. Within each unknown locus, there were no potential secondary SNVs not in linkage disequilibrium (LD) with the lead SNV (*r*^2^ < 0.2) and meeting our look-up significance threshold (*P* < 1 × 10^−5^). Hence only 12 new lead SNVs were carried forward. We also followed-up 12 potential secondary signals at 9 of the 21 previously reported HR loci (further details on selection criteria are provided in the Materials and Methods) (12). None of the selected variants was in LD (*r*^2^ < 0.2) with each other, or with the published SNVs. Thus, a total of 24 variants were taken forward into replication. The UK Biobank dataset provided results for the selected genetic variants (*N* = 134 251 individuals).

**Figure 2:**
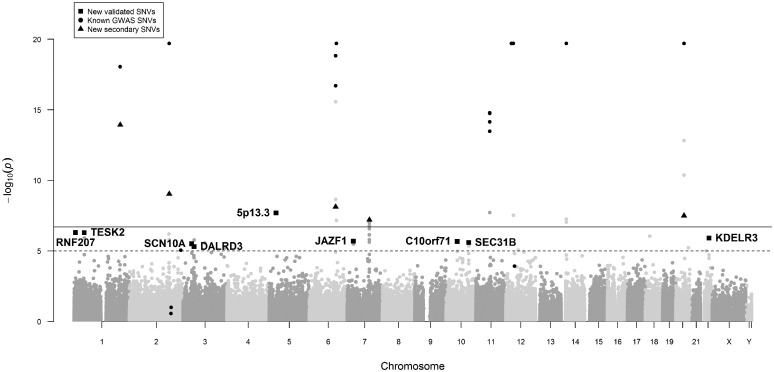
Manhattan plot for the RR-interval discovery meta-analysis in European individuals. The Manhattan plot displays the results from the discovery meta-analysis of RR-intervals from *N* = 104,452 individuals of European ancestry (from 30 cohorts). On the X axis, *P*-values are expressed as −log_10_(*P*) are plotted according to physical genomic locations by chromosome. The Y-axis is truncated to −log_10_(*P*) = 20 with any variants with P < 1 × 10^−20^ displayed on the −log_10_(*P*) = 20 line. The nine novel variants validated from the combined meta-analysis with UK Biobank data are represented by squares. Variants in linkage disequilibrium (LD; *r*^2^ > 0.8) with published GWAS variants are highlighted with black circles (12). New secondary variants validated in our analysis are indicated as triangles. Locus names of the novel loci correspond to the nearest annotated gene, with 5p13.3 denoting an intergenic variant. The dashed line indicates a *P*-value threshold of 1 × 10^−5^, corresponding to the lookup significance threshold and the continuous line indicates a P-value threshold of 2 × 10^−7^, corresponding to exome-wide significance.

Nine of the 12 previously unknown variants were validated based on exome-wide significance (*P* ≤ 2.12 × 10^−7^) in the combined meta-analysis of CHARGE and UK Biobank data, and on Bonferroni-adjusted significance (*P* ≤ 0.0042 for 12 tests) in the replication dataset alone, with concordant directions of effects taking into account the inverse relationship between the RR-interval from the discovery data and HR from the replication data (Table 1; Fig. 2). Indeed, all nine SNV associations were genome-wide significant in the combined meta-analysis (*P* < 5.0 × 10^−8^). Four of our nine validated novel loci were reported in a UK Biobank study (17) that was published after completion of our study (Table 1B). Hence, we present results here for five unreported novel loci (Table 1A;Supplementary Material, Figs S5 and S6).

**Table 1:** Heart rate-associated loci identified from Exome Chip analysis

SNV	Locus	Chr:Pos	EA	EAF	*N*discovery	BETA-RR (SE)	*P*discovery	BETA-HR (SE)	*P*replication	*P*combined
(A) Five unreported novel loci										
rs17853159^a^	*TESK2*	1:45810865	A	0.07	104 452	−6.03 (1.20)	5.02 × 10^−7^	0.31 (0.08)	9.55 × 10^−5^	4.09 × 10^−10^
rs3087866^a^	*DALRD3*	3:49054692	T	0.25	104 452	3.29 (0.72)	4.92 × 10^−6^	−0.31 (0.05)	7.06 × 10^−10^	2.09 × 10^−14^
rs1635852	*JAZF1*	7:28189411	C	0.50	104 452	2.96 (0.62)	2.04 × 10^−6^	−0.15 (0.04)	4.10 × 10^−4^	6.97 × 10^−9^
rs10857472^a^	*C10orf71*	10:50534599	A	0.45	104 452	−2.97 (0.63)	2.11 × 10^−6^	0.16 (0.04)	1.49 × 10^−4^	2.21 × 10^−9^
rs3793706^a^^,b^	*SEC31B*	10:102269085	A	0.22	104 452	3.52 (0.75)	2.54 × 10^−6^	−0.19 (0.05)	2.06 × 10^−4^	3.72 × 10^−9^
(B) Four loci validated in our study and also recently published in the UK Biobank study										
rs709209^a^	*RNF207*	1:6278414	G	0.35	104 452	−3.30 (0.66)	4.94 × 10^−7^	0.27 (0.04)	2.14 × 10^−9^	5.44 × 10^−15^
rs6795970^a^	*SCN10A*	3:38766675	A	0.40	104 452	2.97 (0.64)	3.10 × 10^−6^	−0.24 (0.04)	1.81 × 10^−8^	2.73 × 10^−13^
rs4282331	*5p13.3*	5:30881510	G	0.42	104 452	−3.56 (0.63)	2.03 × 10^−8^	0.26 (0.04)	2.97 × 10^−9^	3.34 × 10^−16^
rs12004^a^	*KDELR3*	22:38877461	G	0.30	104 452	3.30 (0.68)	1.24 × 10^−6^	−0.31 (0.05)	4.92 × 10^−11^	4.04 × 10^−16^

Due to the inverse relationship between R-R interval and HR the opposite beta directions do relate to concordant directions of effect between discovery and replication. SNV, single-nucleotide variant; Chr:Pos, Chromosome:Position based on HG build 19; EA, effect allele; EAF, effect allele frequency from the discovery data; BETA-RR, beta effect estimate of RR-interval (milliseconds) taken from the ExomeRR discovery data; SE, standard error of the effect estimate; *N*, sample size analysed per variant (provided for genotyped discovery data only, as replication data was imputed so *N* = maximum *N* for all variants); BETA-HR, beta effect for heart rate (in beats per minute) taken from the UK Biobank replication data; *P*, *P*-value from either the discovery meta-analysis, the replication data, or the combined meta-analysis of discovery and replication data. Locus name indicates the nearest gene to the HR-associated SNV.

a Indicates that the lead or a proxy SNV (*r*^2^>0.8) is a non-synonymous SNV.

b Indicates if the lead SNV is predicted to be damaging. Mapping to more than 500 kb from either side of a previously reported HR-associated SNV. A novel locus is a genomic region with no SNVs in LD (*r*^2^ < 0.2) with HR-associated SNVs.

Twelve of the 21 HR-associated SNVs from the previously reported GWAS (12) were covered on the Exome Chip, either directly or by a proxy SNV in high LD (*r*^2^ > 0.8). Our discovery meta-analysis showed strong support for the previous findings, with 11 of the 12 SNVs validated at Bonferroni-adjusted significance (*P* ≤ 0.0042 for 12 tests), of which nine were validated at exome-wide significance (*P* < 2 × 10^−7^; Fig. 2). Only rs4140885 at the *TFPI* locus was not supported in our data (*P* = 0.10; Supplementary Material, Table S1).

### Independent secondary signals at known loci

All 12 potential secondary signals at loci previously reported by den Hoed *et al.* (12) were genome-wide significant in the combined meta-analysis (Supplementary Material, Table S2) and are independent to the known SNPs according to LD (*r*^2^ < 0.2). We performed a conditional analysis using Genome-wide Complex Traits Analysis (GCTA) to formally identify secondary signals of association. Five of the 12 validated potential secondary SNVs (within *CD46*, *CCDC141*, *SLC35F1*, *ACHE* and *KIAA1755* loci) were selected within the final GCTA model (Supplementary Material, Table S3). At four of the previously reported HR regions the secondary signals that we identified were confirmed to be statistically independent signals of association: *CD46* (rs2745967), *CCDC141* (rs10497529), *SLC35F1* (rs12210810) and *KIAA1755* (rs41282820) in addition to the known SNV, as both the published SNV and the new secondary SNV were present in the final GCTA model of jointly independent associated variants. Hence, we identified two distinct signals of association at each of these four known HR loci. However, the published SNV at the *ACHE* locus (rs13245899) is not covered on the Exome Chip, or by any proxies (Supplementary Material, Table S1), so the GCTA analysis does not include the known variant. As we are not able to condition on the unavailable published SNV and formally test association jointly with the known SNV, we are unable to statistically confirm the total number of independent signals at the *ACHE* locus.

The secondary SNVs at *CCDC141, ACHE* and *KIAA1755* are non-synonymous variants. Furthermore, the SNVs at *CCDC141* and *KIAA1755* are low-frequency with minor allele frequencies (MAFs) of 3.6 and 1.7%, respectively. Secondary signals have also recently been observed at four of the five loci (*CD46*, *CCDC141*, *SLC35F1* and *ACHE*) in UK Biobank data (17), since completion of our meta-analysis. At *CD46*, our secondary SNV (rs2745967) is in high LD (*r*^2^ = 0.78) with the secondary SNV (rs2745959) reported in UK Biobank, so likely to be the same signal. At *CCDC141* our secondary variant is exactly the same SNV as from UK Biobank (rs10497529). Similarly, at *SLC35F1*, our secondary SNV (rs12210810) is in very high LD (*r*^2^ = 0.98), so is likely to be the same signal. Hence at these three known loci (*CD46*, *CCDC141*, *SLC35F1*), all existing data suggest there are two independent signals of association. At the *ACHE* locus, our secondary SNV (rs542137; ∼38 kb and *r*^2^ < 0.2 from the published SNV) is not in LD (*r*^2^ < 0.2) with the secondary SNV from UK Biobank (rs140367586; ∼659 kb and *r*^2^ < 0.2 from the published SNV). We are unable to clearly determine the number of distinct signals at the *ACHE* locus from our Exome Chip RR-interval discovery meta-analysis data, without the published SNV being covered on the Exome Chip. The low-frequency non-synonymous variant (rs41282820) at the known *KIAA1755* locus is a new, secondary variant, with strong evidence of independent association, it does not overlap with other published findings.

### Variance explained

Twelve of the 21 previously reported HR-associated SNVs (12) covered on the Exome Chip explain 1.14% of RR-interval variance (*P* = 3.96 × 10^−10^) within the 1958 Birth Cohort study (see Materials and Methods). The added contribution of the lead SNVs at our five unreported novel loci, combined with the 12 previously reported SNVs, increases the variance explained to 1.28% overall (*P* = 9.17 × 10^−11^).

### Comparison of results between European and non-European populations

To investigate our data from non-European samples [9358 African Americans (AA), 1411 Hispanic (HIS) and 754 Chinese-Americans (CH); Supplementary Material, Table S4], we first extracted results for the 12 of the 21 previously reported HR-associated SNVs covered on the Exome Chip (12). In contrast to previous results for Europeans, only two known HR-SNVs showed evidence of association (*P* < 0.05), at the *GJA1* and *MYH6* loci, in the AA population only. This is likely due to a lack of power from the smaller non-European sample sizes, considering the power was calculated to be only 48, 11.7 and 8.5% for AA, HIS and CH, respectively. Concordance in the direction of effects compared with Europeans was only significant for AA, with 92, 64 and 50% concordance, corresponding to *P*-values of 2.9 × 10^−3^, 0.16 and 0.23 from binomial tests for AA, HIS and CH, respectively. The lack of support of previous findings from the under-powered non-European data led us to restrict our primary discovery meta-analysis to Europeans only.

We also performed a look-up of the nine validated SNV associations in the non-European samples. Due to the lack of power, and different allele frequencies compared with Europeans, none of the SNVs had results with *P *< 0.05 within any ancestry (Supplementary Material, Fig. S6), and there was little concordance in effect directions: 56% and *P *= 0.246 for AA; 33% and *P *= 0.164 for HIS and CH.

### Gene-based tests

Gene-based testing was performed to identify genes which may have multiple rare variant associations. None of the gene-based test results was significant, after excluding the single most significant low-frequency variant from the tests (Supplementary Material, Table S5).

### Look-up of UK Biobank HR-SNVs

Since completion of our meta-analysis of Exome Chip genotypes, a genome-wide scan for HR has been completed in UK Biobank (17). This study published 46 new HR loci. Four of these novel loci were simultaneously discovered in our analyses (*RNF207*, *SCN10A*, *5p13.3*, *KDELR3:*Table 1B). Among the 42 remaining UK Biobank loci, only five of the lead SNVs were covered on the Exome Chip at *r*^2^ ≥ 0.8. Results from our exome RR European-ancestry meta-analyses show support for all five of these loci (*P* < 0.01; Bonferroni-adjusted significance for five tests; Supplementary Material, Table S6).

### HR loci and association with other traits

To provide insights into possible shared aetiologies or mechanisms of disease, we assessed association of our five unreported novel HR-SNVs (and their proxies, *r*^2^ ≥ 0.8) with other traits. Genome-wide significant phenotype–genotype associations were observed for three novel loci (Supplementary Material, Table S7). The SNV at the *DLRD3* locus was associated with age of menarche. The SNV at the *JAZF1* locus was highly pleiotropic, as shown by associations with several autoimmune disorders (systemic lupus erythematosus, Crohn’s disease and selective immunoglobulin A deficiency), height, type 2 diabetes and *JAZF1* transcript levels in adipose tissue. The SNV at the *SEC31B* locus was associated with plasma palmitoleic acid levels and differential exon expression of *SEC31B*.

### Functional annotation of novel HR-SNVs and candidate genes

Four of the five unreported novel HR-SNVs or their proxies (*r*^2^ > 0.8) are non-synonymous SNVs in *TESK2*, *DALRD3*, *C10orf71* and *SEC31B* (Table 1A). The non-synonymous SNV in *SEC31B* (rs2295774, c.1096T>G, p.Ser332Ala) is in a conserved region of the protein, and is predicted to be damaging using three different algorithms in ANNOVAR (19). We also investigated whether the novel HR-associated SNVs or their proxies (*r*^2^ > 0.8) were associated with changes in expression levels of nearby genes (i.e. as expression quantitative trait loci, or eQTLs) in the Genotype-Tissue Expression database (GTEx) dataset (20). We observed a significant eQTL association at one novel HR locus (Supplementary Material, Table S8). Specifically, the HR increasing allele of the non-synonymous SNV at *SEC31B* was associated with increased levels of *SEC31B* in tibial nerves (*P* = 8.08 × 10^−33^), lung (*P* = 1.22 × 10^−23^), atrial appendage tissue (*P* = 4.56 × 10^−11^) and the left ventricle (*P* = 4.0 × 10^−9^), tissues which may be regarded as physiologically relevant for HR.

**Figure 3:**
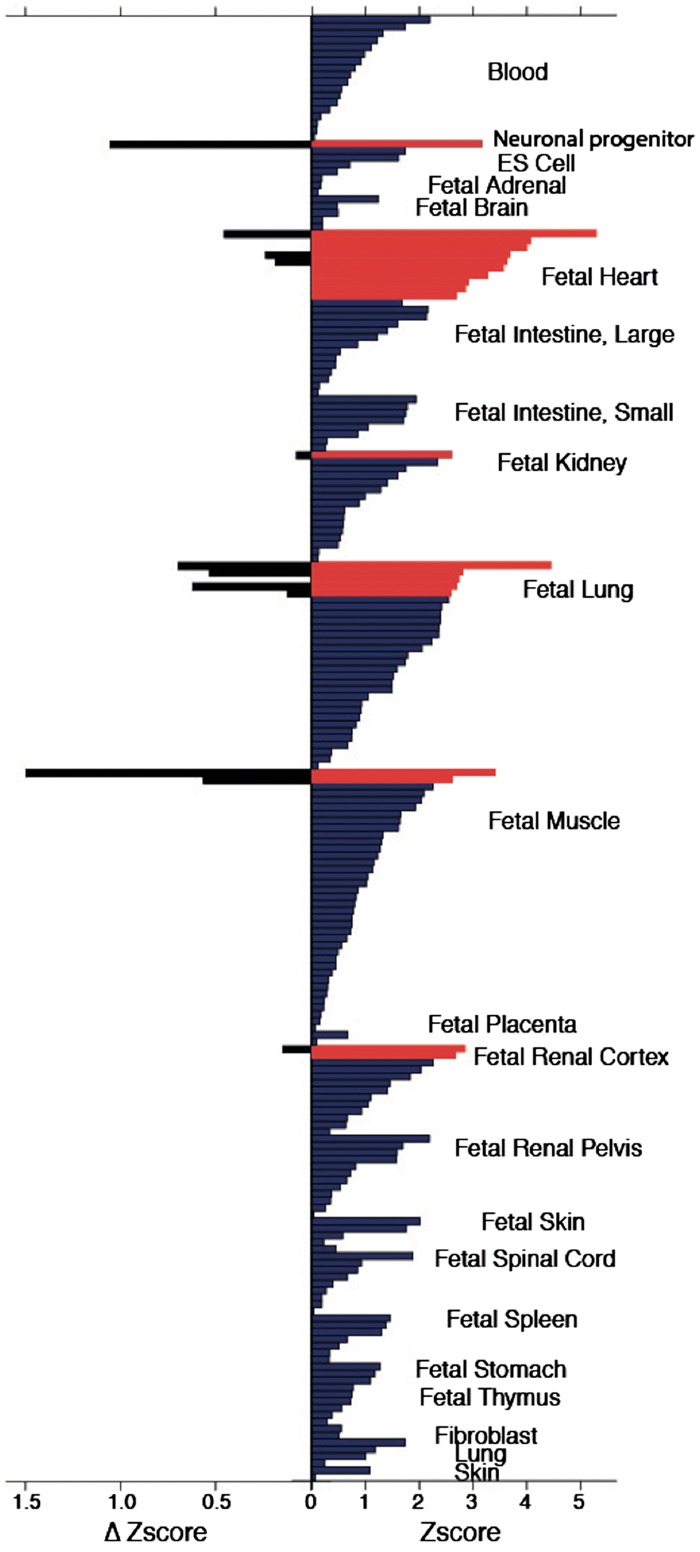
Enrichment of HR-SNVs in DNase I hypersensitive sites of 299 tissue samples. The right panel shows the enrichment of the combined known and novel (all) HR-SNVs in DNase I hypersensitivity sites of 212 Roadmap Epigenome tissue samples (those with positive *Z*-scores). Enrichment is expressed as a Z-score compared with the distribution of 1000 matched background SNV sets. Significant enrichments are shown in red (Z-score ≥ 2.58, false discovery rate (FDR) <1.5%), enrichments below this threshold are shown in blue. The left panel shows the enrichment difference (Δ*Z*score= *Z*score_all_ − *Z*score_known_) for those tissue samples in which we found significant enrichment using all SNPs and that further show a positive change using all SNVs compared with only known SNVs, with increased enrichment hence due to the novel loci identified.

We also observed HR loci to be significantly enriched for DNase I hypersensitive sites (DHSs; Fig. 3). We evaluated regions containing the five unreported novel HR loci and five independent secondary variants at previously reported HR loci (12) together with all 67 published HR-associated SNVs [21 loci reported from the original GWAS (12) plus 46 loci recently published from UK Biobank (17)]. Highest enrichment for DHSs in HR loci occurred within regions that are transcriptionally active in fetal heart tissue and fetal lung, as reported in the UK Biobank study. Moreover, for the first time we found significant enrichment for DHSs in human neuronal progenitor cells (derived from embryonic stem cells) and fetal muscle samples, with the inclusion of our novel loci.

### Pathway analyses

We used Ingenuity pathway analyses to determine whether there was any increased enrichment in HR-associated pathways with the contribution of our five newly identified loci. We identified 16 significantly enriched pathways at *P* < 1 × 10^−4^. Most of these pathways are related to the cardiovascular system and involve, for example, supraventricular arrhythmias, dilated cardiomyopathy and HR (Supplementary Material, Table S9).

### Coding variants at HR loci

The Exome Chip provides a unique opportunity to search for coding variants within known HR loci. Although GWAS analyses typically identify intron or intergenic variants, Exome Chip analysis may identify HR-associated coding variants, which would point to candidate causal genes. We considered all 67 published HR loci [21 previously reported GWAS loci (12) plus 46 recently published loci from UK Biobank (17)] and extracted all SNVs in high LD with the lead variants (*r*^2^ ≥ 0.8), tagging the same association signal, restricted to variants covered on the Exome Chip. We further filtered variants to obtain SNVs that reached exome-wide significance for associations with RR-interval in our primary discovery meta-analysis, to ensure that variants have a highly significant association with the trait. Coding SNVs were identified, using the CHARGE Exome Chip annotation file.

We only observed two such coding variants in two reported loci: *CCDC141* and *KIAA1755*. The published *CCDC141* coding variant was previously annotated as being non-synonymous (12), and is predicted to be damaging in our annotation (rs17362588; p.Arg935Trp). The coding SNV at *KIAA1755* is the best proxy (*r*^2^ ∼ 1) for the published non-synonymous SNV (rs6127471) covered on the Exome Chip (Supplementary Material, Table S1). The original GWAS (12) had reported this signal as non-synonymous. Therefore, our Exome Chip analyses do not reveal any new evidence of likely causal coding variants at well-established HR loci.

### Regulatory variants at HR loci

Our analyses of coding variants at all known HR loci indicated that the majority of HR-associated SNVs and the variants in high LD with them are non-coding. We thus investigated which variants could have a causal effect through regulatory chromatin interactions, such as promoter–enhancer contacts. We considered all 67 published HR loci [21 previously reported GWAS loci (12) plus 46 recently published loci from UK Biobank (17)], and the five novel loci reported here. We found variants that potentially affect enhancer function using RegulomeDB (21) and found genes whose promoter regions form significant chromatin interaction with them from right ventricle Hi-C data (22). We found 64 potential target genes in 49 HR loci (4 new loci, 18 loci from the GWAS and 27 loci from the UK Biobank study; Supplementary Material, Table S10). Including these long-range interactors in the candidate causal genes list increased the significance of enrichment for many HR-related terms, such as arrhythmia and cardiac fibrillation in our Ingenuity® Pathway Analysis (IPA®; Supplementary Material, Table S11).

For newly identified loci, the *TESK2* promoter had a long-range interaction with the SNVs with highest regulatory potential in the locus, underlining it as a candidate. *LOC441204*, a gene of unknown function was found to interact with the *JAZF1* locus. At the *SEC31B* locus, there were interactions with two genes, *SCD* and *SLF2*. At the *C10orf71* locus, *MAPK8* showed the most significant interaction.

In the 21 loci from the previously published GWAS (12), we identified significant chromatin contacts for the regulatory SNVs of 18 loci. We found *CALCRL*, *TTN*, *HTR2B*, *PLD1* and *CHRM2* as strongest interactors at the *TFPI*, *CCDC141*, *B3GNT7*, *FNDC3B* and *CHRM2* loci, respectively, out of these only *CALCRL* is in LD (*r*^2^ > 0.8) with the lead SNV. The previous study (12) functionally tested 31 candidate genes, they found 20 of them to have an HR phenotype in either *Drosophila melanogaster* or *Danio rerio* experiments. All five of the strongest interactor genes were amongst the 20 genes with an HR phenotype.

Finally, we found 41 potential causal genes that have not been implicated by previous GWASs. A few of these genes have a cardiac function, including *RAPGEF4* (18) and *PIM1* (23), whereas some are involved in neuronal development and function, e.g. *PBX3*, *NRNX3*. These candidates open up new avenues that may aid our understanding of HR biology.

## Discussion

Our meta-analysis of Exome Chip genotypes yielded five unreported novel HR loci, and one unreported independent new secondary signal, which was a low-frequency non-synonymous SNV at the previously reported *KIAA1755* locus. Our data strongly supported the association of SNVs at 11 of the 12 previously reported GWAS loci that were covered on the Exome Chip. All lead SNVs at all validated novel loci are common (MAF ≥ 5%) and have similar effect sizes, which are smaller than the effect sizes for the majority of previously reported SNVs (Supplementary Material, Fig. S7). Our study did not yield any rare SNV associations with HR, indicating that much larger sample sizes will be required in future studies to have sufficient power to detect effects of any rare variants and assess their contributions to HR heritability.

The same observation of the need of larger sample sizes applies to the analysis of HR loci identified within Europeans in other ancestries, where the lack of significance and concordance in the results from non-European populations is most likely due to a lack of power, as well as differences in the allele frequencies and LD patterns between Europeans and non-Europeans. As the non-European samples were much smaller, we did not perform a comprehensive comparison across populations or a robust trans-ethnic meta-analysis.

Annotation of novel HR-SNVs or their close proxies, eQTL analyses and long-range chromatin interactions in heart tissue reveal new potential causal candidate HR genes (Supplementary Material, Tables S10 and S12). At the *SEC31B* locus there is a predicted damaging non-synonymous variant in *SEC31B*, and SNVs at this locus are also significantly associated with *SEC31B* expression levels. Although its precise function is unknown, the *SEC31B* gene encodes SEC31 homolog B, a COPII coat complex component. SEC31B has been proposed to function in vesicle budding, and cargo export from the endoplasmic reticulum (24). The gene is ubiquitously expressed at low levels, but there are higher levels of expression in the cerebellum. There are 13 transcripts, and thus several predicted SEC31B proteins. The major isoform is 129 kDa, but the HR-associated non-synonymous SNV maps to all *SEC31B* transcripts. There are no existing mouse models, and the predicted protein does not directly interact with other proteins or pathways currently recognized as being important to HR. Chromatin interactions in heart tissue indicate *SCD* and *SLF2* as two other candidate genes for consideration at this locus. *SCD* encodes a stearoyl-CoA desaturase, which has a role in myocardial dysfunction (25) and *SLF2* encodes the SMC5–SMC6 complex localization factor 2. *TESK2*, *C10orf71* and *DALRD3* can be considered as candidates for further analyses, based on the lead SNVs being non-synonymous variants in each gene. *TESK2* encodes a serine/threonine protein kinase with an N-terminal protein kinase domain that is structurally similar to the kinase domains of testis-specific protein kinase-1 and the LIM motif-containing protein kinases. *TESK2* is ubiquitously expressed, but its function is unknown (26). There is also support for *TESK2* from the chromatin interaction analyses. *C10orf71* encodes an open reading frame of unknown function that is highly expressed in heart and skeletal muscle. Chromatin interaction analyses indicate *MAPK8* as a second candidate gene at the *C10orf71* locus, *MAPK8* is involved in formation of the heart as well as HR regulation (27,28). *DALRD3* encodes a protein with a DALR anticodon-binding domain similar to that of class Ia aminoacyl tRNA synthetases (29).

The conditional analysis results provided one new, unreported association at a previously reported HR locus, *KIAA1755* (rs41282820; c.1528C>T or c.1528C>A; p. Arg510Ter, a loss of function variant). *KIAA1755* is predicted to encode an uncharacterized protein, and is only characterized at the transcriptional level. The transcript is highly expressed in the brain and nerves, and it is also expressed in the heart.

Our analyses and the recently published UK Biobank analyses (17) discovered a second low-frequency non-synonymous SNV at *CCDC141* (rs10497529, c. 442C > T, P. Ala141Val). *CCDC141* (also known as *CAMDI*) encodes the coiled-coil domain containing 141 protein and interacts with DISC1 (disrupted in schizophrenia 1) and MYL2 (phosphorylatable myosin light chain). *CCDC141* is highly expressed in heart muscle (30). Knockdown of CCDC141 in neurons leads to abnormal cortical neuronal migration, but there are otherwise limited functional studies of CCDC141 (30). The *CCDC141* locus includes *TTN* (titin), which encodes a major structural protein in striated muscle. *TTN* mutations are associated with a range of hereditary myopathies (31). Prior work (12) using RNA interference in *Drosophila melanogaster* has shown that knockdown of *TTN* leads to significant changes in resting HR and HR post tachypacing, supporting *TTN* is a causal candidate gene at this locus. The new data described here implicate *CCDC141* as a second candidate gene at this locus for functional follow-up.

Enrichment analysis of HR variants in DNase I hypersensitivity sites across nearly 300 tissue samples and cell lines indicated new candidate tissues, such as neuronal progenitors and fetal muscle as being functionally relevant. Our data suggest these tissues should be targeted for future functional studies.

Our long-range regulatory chromatin interaction analyses provided additional support for some of the candidate genes have been experimentally tested previously (12) and shown to have an HR-related phenotype (*CALCRL*, *TTN*, *HTR2B*, *PLD1* and *CHRM2*). By expanding the list of HR loci to include new and published, several new candidate genes are highlighted for functional studies in Supplementary Material, Table S10.

The Exome Chip contains non-synonymous, splicing and stop-coding variants that are thought to alter protein expression and function. Our analyses discovered four novel coding variants, indicating potential candidate causal genes at these loci. Our two-stage study design permitted the robust validation of all our novel loci findings, with a large replication sample size from UK Biobank (*N* = 134 251) to add together to our European discovery data (*N* = 104 452) for a large combined meta-analysis. However, due to the Exome Chip covering mainly coding regions, we were not able to compare results with all previous GWAS findings. In conclusion, our results taken together with recent studies (12) indicate HR-associated SNVs are mostly common (MAF > 5%) and have relatively small effect sizes. The maximum effect sizes reported thus far are ∼0.70 BPM per allele and MAF of 1% for SNVs at *CCDC141* (rs17362588) and *GJA1* (rs1015451). An analysis of much larger sample sizes (1M and above) including rare and common SNVs, and samples across different ancestries may provide further information on the contributions of both coding and non-coding variants, and the importance of rare coding variants in HR.

## Materials and Methods

### Study populations, phenotypes and exclusions

Thirty cohorts contributed data to the discovery meta-analysis in individuals of European ancestry. Details of all participating cohorts are provided in Supplementary Material, Table S13, including phenotype, cohort ancestry, study design and key references. The UK Biobank study, which was only recently published since the completion of our meta-analysis (17), provided results for replication analyses. Details of this study are also included in Supplementary Material, Table S13.

All participating cohorts either measured RR-intervals from the standard 12-lead electrocardiogram (ECG) or used HR measurements (in beats per minute) from peripheral pulse measurements (Supplementary Material, Table S14), which were converted to the RR-interval scale (in milliseconds) using the inverse relationship formula: RR (ms) = 60 000/HR (BPM). The discovery analysis was undertaken using the RR-interval phenotype. The exclusion criteria included: extreme RR-intervals (< 600 or > 1500 ms), atrial fibrillation on the ECG, a history of myocardial infarction or heart failure, use of non-dihydropyridine calcium-antagonists [Anatomic Therapeutic Chemical (ATC) code C08D], digoxin (ATC code C01AA5), second or third degree atrioventricular block and a pacemaker signal on the ECG. Local ethics committees approved the contributing studies from the CHARGE consortium, and all individuals provided their consent in writing. The UK Biobank study has approval from the North West Multi-centre Research Ethics Committee and has Research Tissue Bank approval.

### Study-level genotyping and quality control

All discovery cohorts were genotyped using a human Exome Chip array (exact details of the chip for each study are provided in Supplementary Material, Table S15). Quality control (QC) was done according to CHARGE Exome QC guidelines, including joint variant calling with zCall (32). At the study-level, the sample-level QC consisted of excluding samples of non-European ancestry (for European-ancestry cohorts), samples with call rates <95%, samples with sex discordance or related samples with an unexpected high identical by descent estimate. It was recommended that principal components (PCs) be obtained using variants with MAF ≥ 1%. The variant QC consisted of exclusion of SNVs with call rate < 95%, with Hardy–Weinberg equilibrium values of *P* < 1 × 10^−6^, and of variants that were strongly associated with plate assignment.

### Study-level statistical analysis

Each cohort performed two SNV association analyses using an additive model implemented with the R package *SeqMeta*, http://cran.r-project.org/web/packages/seqMeta/index.html. Analyses were stratified by ancestry. One SNV association analysis used an untransformed model with RR-interval as the outcome, adjusted for age, sex, BMI and cohort-specific adjustments. The other SNV association analysis was a model based on the rank-based inverse-normal transformed residuals (RR-INVN), with residuals taken from a linear regression RR-interval adjusted for age, sex and BMI covariates. The RR-INVN analysis was performed to check for potential sensitivity to deviations from normality within the analysis of rare variants. Additional cohort-specific covariate adjustments were also applied, which included for example PCs or family structure.

### Central QC and meta-analyses

We performed additional QC checks centrally. For each study, we checked the sample size and the total number of SNVs (monomorphic and polymorphic) and assessed the beta distribution. Within each cohort’s results, all monomorphic SNVs were checked to have non-available results. In order to detect potential strand-flip issues, the cohort-coded effect allele frequencies (EAF) of each SNV were compared with the meta-analysed EAF of a group of CHARGE cohorts (AGES, ARIC, CHS, FHS and WHI). Any discordant SNVs showing cohort-EAF ∼ 0 in at least one study, but meta-EAF ∼ 1, or vice versa, were excluded from the central meta-analysis. A set of approximately 11 000 SNVs that were known to have QC issues from central CHARGE QC were also excluded from the meta-analysis. Quantile–Quantile plots were produced to inspect each cohort. After all QC steps were completed 235 677 SNVs remained. The results from all cohorts were then combined into a discovery meta-analysis using the *SeqMeta* R package.

### Sensitivity analyses

A sensitivity analysis was performed on the use of beta-blockers (ATC code C07) due to the recognized effects of beta-blockers on HR. All cohorts with data on beta-blocker use were re-analysed with exclusion of individuals using beta-blockers at the time of phenotype measurement. Results of this meta-analysis were compared with the results from the same subset of cohorts with beta-blocker users included.

### Selection of variants for replication

All SNVs with *P* < 1 × 10^−5^ from the discovery meta-analysis in European individuals were considered for follow-up. As a QC step after meta-analysis, we excluded four SNVs with unrealistically high beta values, large standard errors and results that were reported in less than four studies. We defined a novel locus as a genomic region (i) with SNVs not in LD (*r*^2^ < 0.2) with any well-established HR-associated SNVs from the previously reported GWAS (12) (Supplementary Material, Table S1), and (ii) mapping to more than 500 kb from either side of a previously reported HR-associated SNV. At the time of our study, there were 21 loci reported from GWAS analyses with HR-associated SNVs (12). A potential secondary signal within a previously reported locus was defined as being within a 1 Mb region centred around the published SNV, but not in LD (*r*^2^ < 0.2) with the published SNV in that region. LocusZoom plots were produced for all selected SNVs. Only the lead SNV was carried forward, for each signal being followed up. Specifically, the most significantly associated SNV was selected for any SNVs in pairwise-LD (*r*^2^ > 0.2). LD was calculated within UK Biobank genetic data, in order to calculate pairwise-LD for all 21 known SNVs (not only those covered on the Exome Chip).

### Replication analyses

We used data from UK Biobank for replication of the selected SNVs (at the time of analysis genetic data were available for 150 000 individuals). The UK Biobank data were analysed with untransformed HR as the phenotype, with no exclusions for medication use. In UK Biobank resting HR was assessed by two methods: first, pulse rate using an automated reading during blood pressure measurement, and second, pulse rate during arterial stiffness measurement using the pulse wave form obtained of the finger with an infra red sensor. When multiple HR measurements were available during the first visit for an individual, these measurements were averaged. In 99.7% of participants at least one single measurement was available. Individuals were excluded with extreme (> 4 SD) values (*N* = 818). Further details are provided (17). The results of our European exome discovery meta-analysis for RR were combined with the UK Biobank replication results for HR (*N* = 134 251), and a combined meta-analysis, using sample-size weighted fixed effects meta-analysis in METAL was performed (33). All alleles were aligned between the discovery and replication data, and the inverse relationship between RR-interval and HR was taken into account, i.e. so that a negative beta direction from our discovery data for a decreased effect on RR-interval was made equivalent to a positive beta.

A novel locus was declared if the lead SNV reached exome-wide significance in the combined meta-analysis of discovery and replication data (*P* < 2.12 × 10^−7^) and replicated with Bonferroni-adjusted significance (*P* < 0.0042 for 12 tests) in the replication data alone. In addition, the directions of effect between the discovery and replication data were required to be concordant, taking into account the inverse relationship between RR from our discovery data and HR from the replication data.

Potential secondary SNVs at known regions were declared as validated if there was an exome-wide significant association in the combined meta-analysis. Variants that validated were subsequently tested for independence from previously reported HR variants in a conditional analysis.

### Conditional analysis

In order to determine whether the validated secondary signals at previously reported loci were independent of the published SNV, conditional analysis was performed within GCTA software (34) applying the –cojo method (consisting of conditional and joint analysis with stepwise model selection). The input data were the exome-wide summary statistics from the full discovery meta-analysis of RR-interval in Europeans. The 1958 Birth Cohort Study (1958BC; *N* = 5815) dataset was used as the reference for genotype data, because it represents one of the largest discovery studies (See Supplementary Material, Table S13). LD was calculated between pairwise SNVs, but any SNVs further than 10 Mb apart were assumed to not be in LD. All autosomal chromosomes were analysed, with MAF restricted to ≥ 0.01%, to allow for low frequency secondary SNVs, whilst taking into account the statistical power achievable. To allow for secondary associations a *P*-value cut-off of 1 × 10^−4^ was used as the modelling selection threshold within the GCTA analysis. Results were then extracted for the nine previously reported regions, within which potential secondary signals had been validated from the combined meta-analysis. To be consistent with the look-up threshold for selecting SNVs to carry forward from discovery to replication, results were restricted to SNVs with a significance level of *P* < 1 × 10^−5^ from both the discovery meta-analysis and the joint association from GCTA.

### Gene-based testing

Gene-based testing was conducted using the primary discovery data in Europeans. Analysis was performed using the SNV-set Kernel Association (SKAT) test within the *seqMeta* R Package. SKAT tests were performed according to two different MAF filters of 1% and of 5%, and three different levels of variant filtering, based on annotations within the CHARGE Exome SNP Info annotation file: (i) all variants, (ii) variants deemed predicted to be damaging (24) and (iii) variants that were non-synonymous or leading to abnormal splicing. For gene-based tests we adjusted for multiple testing using the Bonferroni correction, according to the number of genes tested. The gene-wide significance level was calculated as 1.98 × 10^−6^ for 25 241 tests (i.e. the number of genes on the Exome Chip). For any genes attaining significance, the gene-based tests were repeated with exclusion of the most significantly associated lead variant, in order to confirm that the association was due to multiple rare variants.

### Non-European ancestry analyses

Association results were also received for non-European samples. Analysis and QC were performed as described for the European data. A meta-analysis was performed centrally in seqMeta for AA ancestry, combining data from the five AA cohorts. Study-level results remained for HIS and CH ancestries (from only the MESA cohort), in order to consider the three non-European ancestries (AA, CH and HIS) separately from stratified analyses. Due to the smaller sample sizes, power calculations were performed using the Genetic Power Calculator (35), based on the average percent trait variance explained per locus being 0.04%, according to the recently published results from 64 validated HR loci explaining ∼2.5% of HR variance (17). To assess the level of heterogeneity by ancestry in non-European data, we performed a look-up of SNVs at the 12 published HR loci covered on the Exome Chip, extracting results for these variants from each of the AA, CH and HIS results. We restricted our primary discovery analysis to Europeans only after finding a lack of significant validation and concordance between EUR and non-EUR data for previously reported HR variants. As a secondary analysis, we performed look-ups of all validated novel loci within the non-European data. The forest plots for all validated novel loci display non-European results, to serve as a comparison to results within Europeans. In addition to calculating the percentage of concordance of effect directions for each ancestry compared with Europeans, a Binomial sign test was also performed in R. This test was based on the number of SNVs with consistent effect directions, and it was done to determine whether the concordance was higher than expected by chance alone, using *P *< 0.05 to declare significant concordance.

### Variance explained

The percentage variance explained for RR-interval was calculated using data from all subjects in the 1958BC study. The SNV genotypes were extracted from the 1958BC Exome Chip data and considered in two different sets: the 12 previously reported SNVs covered on the chip including proxies (*r*^2^ ≥ 0.8; see Supplementary Material, Table S1); and the lead SNVs from the five unreported novel loci (see Table 1A). First, RR-interval was regressed in a linear model against the sex and BMI covariates (not age, as all 1958BC subjects are of same age). Then the trait residuals from this first model were used as the phenotype in a second linear regression model, with all SNVs in the given set analysed jointly as multiple predictors, and adjusted for the top 10 PCs. The percentage trait variance explained by the set of SNPs was estimated from this second model, according to the adjusted *R*^2^ value.

### HR loci annotation

For the purposes of annotation, all signals were expanded to include SNVs in LD. LD was calculated within the UK Biobank full genetic dataset using PLINK (v1.9). All variants with an *r*^2^ ≥ 0.8 within 500 kb downstream or upstream of the SNVs of interest were identified. These variants were annotated using ANNOVAR [vJun2015 (19)]. ANNOVAR functionally annotates variants, provides their conservation score, identifies SNVs that may cause protein-coding changes and reports their damaging prediction scores. Various prediction scores are available in ANNOVAR, including SIFT, PolyPhen and MutationTaster, among others.

We investigated the unreported novel SNVs and their proxies (*r*^2^ ≥ 0.8) across 44 tissues available in the GTEx dataset (20) for eQTLs. We reviewed the results for SNV-eQTL associations across all tissues, focusing on the heart, nerve, lung, muscle, adrenal and brain tissues which may be relevant tissues for HR based on known physiology of HR and our results from the enrichment analysis. Genes reported as eQTLs are based on study-specific significance thresholds (*P*-values < 10^−8^) and *r*^2^ ≥ 0.8 between HR-SNV and top-eQTL SNV (the SNV most significantly associated with transcript).

### PhenoScanner

PhenoScanner (36) was used to identify variants that are associated with other traits. All proxy SNVs in high LD (*r*^2^ ≥ 0.8) with the lead SNVs at our five unreported novel loci were investigated in the PhenoScanner 1000 Genomes reference dataset. Results were filtered to those reaching a genome-wide significance *P*-value ≤ 5 × 10^−8^.

### Potential candidate genes at new HR loci

Candidate genes at each locus were compiled using LD information, ANNOVAR-derived annotation and eQTL lookup results. A literature review was conducted for potential candidate genes at each new HR locus. Sources of information included: published articles, GeneCards, Online Mendelian Inheritance in Man®, the Human Protein Atlas, STRING and UniProt. We searched for information on the corresponding mouse models via the International Mouse Phenotyping Consortium and the Jackson Laboratory online catalogue. URLs for each of the sources is provided in the URL section below.

### Pathway analyses

Pathway analyses were performed using QIAGEN’s IPA^®^ (QIAGEN Redwood City) software. In order to distinguish the pathway enrichment contribution of novel loci from known HR loci, two sets of analyses were conducted. The first analysis captured the total known signal to date, investigating all 67 loci currently published, which include the 21 loci from the previously reported GWAS (12) and the 46 loci recently published from UK Biobank (17) since the completion of our meta-analysis. The second analysis included our five unreported novel loci in addition to all the previously reported loci. In each case, the analysis included all genes annotated from the lead SNVs and their proxies (*r*^2^ ≥ 0.8). Results were filtered for pathway enrichment of *P*-values ≤ 10^−4^. We specifically report the pathways for which enrichment is increased with the inclusion of genes from our novel loci.

### Enrichment in DHSs

To identify the tissues in which HR-associated SNVs are active, we used FORGE to look for enrichment of DHSs in 299 tissue samples from the Roadmap Epigenome Project (37). FORGE calculates enrichment for overlap of HR variants with DHS by comparison with overlap of DHSs with 1000 matched background variant sets (matching distance to transcriptional start sites, GC content and MAF).

We performed two different enrichment analyses. First, we did a ‘known’ analysis using all 67 currently published lead SNVs to date [21 previously reported from the original GWAS (12) and 46 new loci from the recently published UK Biobank study (17)]. Second, we did an ‘all’ analysis using the lead SNVs at our five unreported novel loci and the five independent secondary SNVs that we found at previously reported loci; together with the 67 known signals, denoted as the ‘all’ analysis. We compared the enrichment results of the two analyses, in order to identify any new enrichment due to the inclusion of our novel loci. The enrichment is expressed as *Z*-score statistics. A *Z*-score of 2.58 was used as a threshold for statistical significance, which corresponds to false discovery rate (FDR) < 1.5%. We calculated the *Z*-score_all_ − *Z*-score_known_ (Δ*Z*-score) for those tissue samples that were found statistically significant in the ‘all’ analysis in order to assess the effect of the 10 new, additional SNVs from our study.

### Regulatory potential of SNVs

We selected the HR-associated SNVs and proxies in LD (*r*^2^ ≥ 0.8; calculated using the UK Biobank full genetic dataset) that were identified in this study, and from the previous GWAS (16) and UK Biobank studies (17) for annotation. To identify the potential regulatory variants, we retrieve the functional confidence score for SNVs from the RegulomeDb database (21). RegulomeDb assigns a functional confidence score to each SNV by overlapping them with functional genomic data mainly from ENCODE (e.g. DNase I hypersensitivity, DNase I footprinting, ChIP-seq), with eQTL data and with computational prediction (e.g. TF-binding sites and their disruption). We considered any SNP with at least one functional annotation to have regulatory potential (this corresponds to functional confidence scores: 1a-6).

### Long-range regulatory contacts

Using significant long-range chromatin interactions as identified by Fit-Hi-C in right ventricle Hi-C data [40 kb resolution (22)], we annotated the potential regulatory SNVs with potential target genes, whose promoter is in contact with the given SNV. Where the 40-kb genomic region containing the SNV had more significant promoter interactions, we show the genes in order of most significant interaction to least significant. For every locus, we took the gene that had the most significant promoter interaction with a regulatory SNV, and using IPA®, we assessed which pathways were affected, and specifically those that were enriched compared to using only genes in LD with HR-SNVs.

## Supplementary Material

Supplementary Material is available at *HMG* online.

## Supplementary Material

Supplementary Data
