# Supplementary material for: Discovery of novel heart rate-associated loci using the Exome Chip
Source: Hum Mol Genet. 2017 Apr 3;26(12):2346–63. doi: 10.1093/hmg/ddx113 (PMC5458336; doi:10.1093/hmg/ddx113)
Supplement: Supplementary Data [file ddx113_Supp.zip › van den Berg_Exome_Chip_Heart Rate_Suppl Material_Revision_12_03_2017_CLEAN.docx]

**Discovery of novel heart rate-associated loci using the Exome Chip**

***van den Berg et al.***

**Supplementary Material**

**1. Supplementary Figure Titles**

**Supplementary Figure 1**: Quantile-Quantile (QQ) plot of the discovery phase meta-analysis of RR-interval among the European-ancestry individuals.

**Supplementary Figure 2: (a**) Comparison of the betas between the untransformed RR-interval meta-analysis and the inverse normalized RR-interval residuals (RR-INVN) meta-analysis; (**b**) Comparison of the *P*-values between the untransformed RR-interval meta-analysis and the inverse normalized RR-interval residuals (RR-INVN) meta-analysis.

**Supplementary Figure 3:** **(a)** Histogram showing the distribution of RR-interval phenotype in the 1958BC study; **(b)** Quantile-Quantile (QQ) plot of RR-interval phenotype in the 1958BC study.

**Supplementary Figure 4: (a)** Comparison of the betas between the RR-interval meta-analysis with and without beta-blocker users; **(b)** Comparison of the *P*-values between the RR-interval meta-analysis with and without beta-blocker users.

**Supplementary Figure 5**: Locus Zoom plots for the five unreported novel loci.

**Supplementary Figure 6**: Forest plots showing the associations of the five unreported novel loci within all European discovery cohorts and non-European studies.

**Supplementary Figure 7**: Effect sizes of heart rate associated variants: Comparison between novel loci and previously reported loci.

**2. Supplementary Tables**

**Supplementary Table 1:** Results in our European dataset for twenty-one previously reported heart rate loci

**Supplementary Table 2:** Validated potential secondary associations at previously reported heart rate loci

**Supplementary Table 3:** Results from conditional analysis using GCTA for previously reported heart rate loci

**Supplementary Table 4:** Associations of 12 previously reported heart rate loci in non-European cohorts

**Supplementary Table 5:** Gene-based analysis results

**Supplementary Table 6:** Look-up results from Exome RR European discovery meta-analysis for recently published loci from the UK Biobank study

**Supplementary Table 7:** Novel heart rate loci associations with other traits using PhenoScanner

**Supplementary Table 8:** Expression quantitative trait locus (eQTL) analysis of the novel heart rate loci

**Supplementary Table 9:** Pathway Enrichment Analysis

**Supplementary Table 10:** Long-range interactors

**Supplementary Table 11:** Pathway enrichment including long-range interactors

**Supplementary Table 12:** Annotation of novel heart rate loci and information on candidate genes

**Supplementary Table 13:** Cohort study information

**Supplementary Table 14:** Descriptive summary statistics within each cohort

**Supplementary Table 15:** Genetic information for each cohort

**Supplementary Figures**

**Supplementary Figure 1**: Quantile-Quantile (QQ) plot of the discovery phase meta-analysis of RR-interval among the European-ancestry individuals


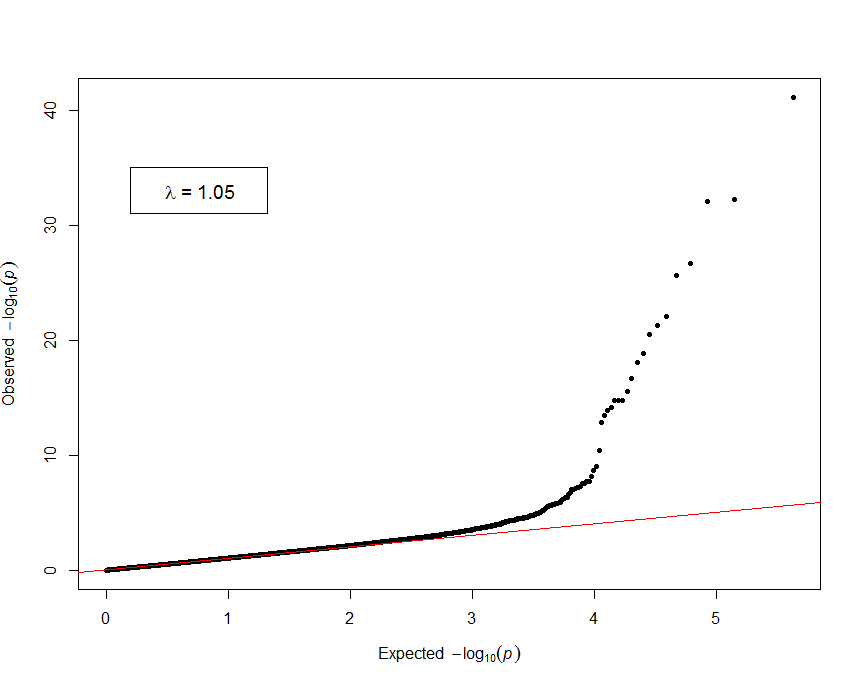


The red line is the line Y = X. The lambda indicates the genomic inflation factor. P-values are plotted on a –log10 scale.

**Supplementary Figure 2**

**a**: Comparison of the betas between the untransformed RR-interval meta-analysis and the inverse normalized RR-interval residuals (RR-INVN) meta-analysis


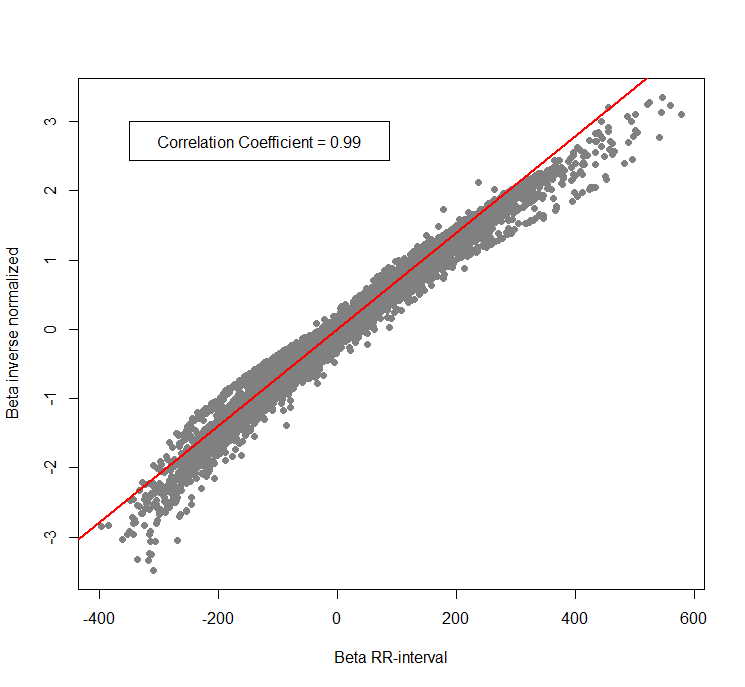


Betas for RR interval (ms) are based on the meta-analysis of the 30 European-ancestry cohorts (N = 104,452). The red regression line is based on a linear regression with outcome Beta(RR-INVN) and determinant Beta (RR-interval). The Pearson’s correlation coefficient is indicated.

**b**: Comparison of the *P*-values between the untransformed RR-interval meta-analysis and the inverse normalized RR-interval residuals (RR-INVN) meta-analysis


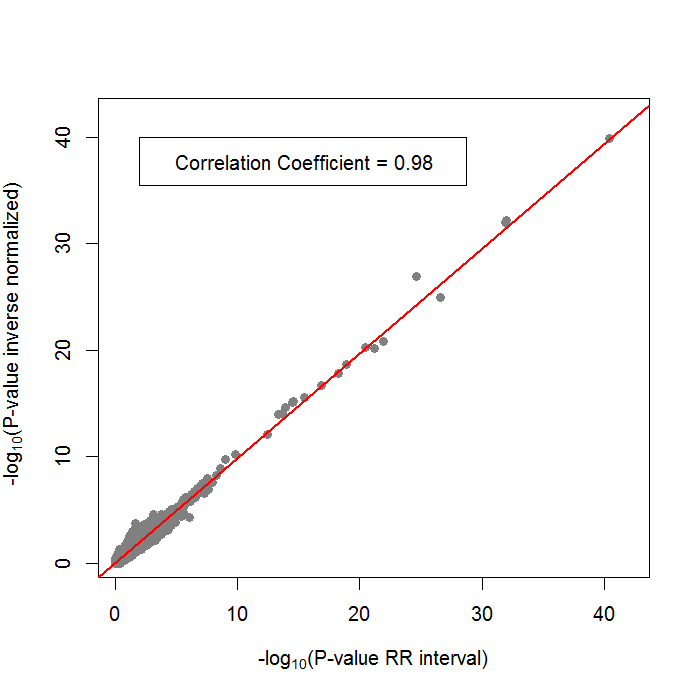


P-value results are based on the meta-analysis of the 30 European-ancestry cohorts (N = 104,452). The red regression line is based on a linear regression with outcome *P*-value (RR-INVN) and determinant *P*-value (RR-interval). The Pearson’s correlation coefficient is indicated.

**Supplementary Figure 3:**

1. Histogram showing the distribution of RR-interval phenotype in the 1958BC study

The normal distribution with the same mean and standard deviation as the data is shown in blue, for RR interval (ms).

1. Quantile-Quantile (QQ) plot of RR-interval phenotype in the 1958BC study

The ranked RR-intervals (in milliseconds) of the 1985BC study are plotted against a ranked standard normal distribution.

**Supplementary Figure 4**

**a**: Comparison of the betas between the RR-interval meta-analysis with and without beta-blocker users.


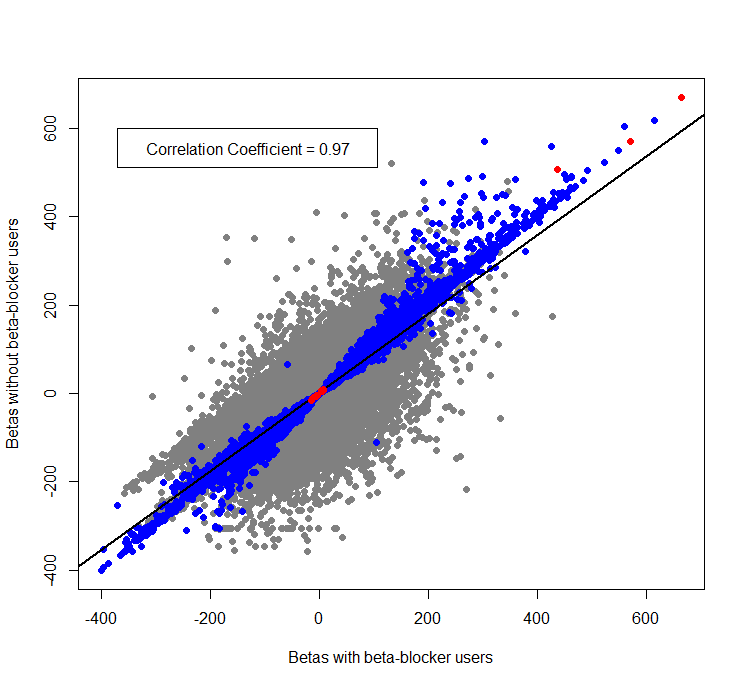


The beta results plotted are for RR interval in milliseconds. The blue points indicate SNVs with *P* values below 0.05; Red points indicate SNVs with *P* values below 1 x 10^-5^, according to *P*-values from the final primary EUR discovery meta-analysis. The reported correlation coefficient is based on the SNVs with *P* value below 0.05 from the final primary European discovery meta-analysis. The three red points in the top right corner were excluded from the meta-analysis results after quality control due to their large betas and standard errors. The results for the meta-analysis with beta-blocker users included were taken from data from the same 17 cohorts as those with beta-blocker users excluded: 1958BC, AGES, ASCOT-UK, ASCOT-SC, BRIGHT, CHS, ERF, GODARTS, Generation Scotland, KORCULA, UHP, MGH, NEO, RS, SardiNIA, SHIP, YFS (N=48,347).

**b**: Comparison of the *P*-values between the RR-interval meta-analysis with and without beta-blocker users.


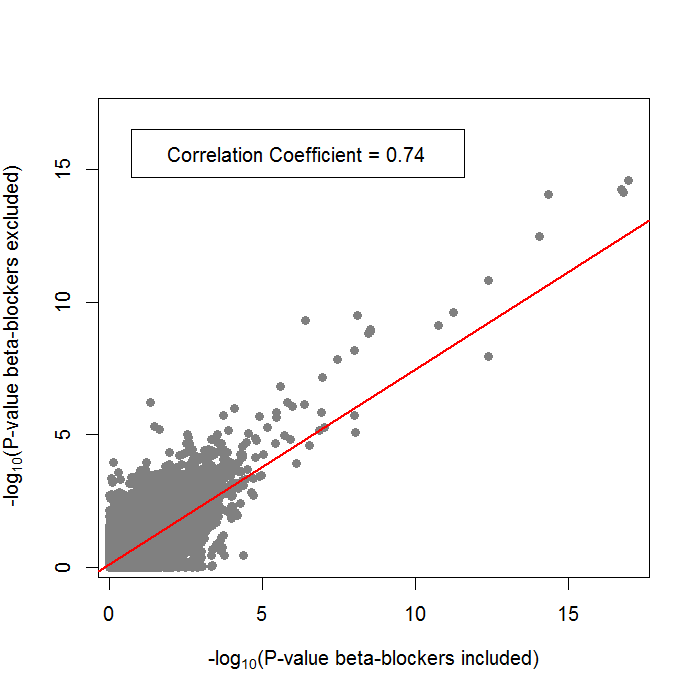


The results for the meta-analysis with beta-blocker users included were taken from data from the same 17 cohorts as those with beta-blocker users excluded: 1958BC, AGES, ASCOT UK, ASCOT SC, BRIGHT, CHS, ERF, GODARTS, Generation Scotland, KORCULA, UHP, MGH, NEO, RS, SardiNIA, SHIP, YFS (N = 48,347)

**Supplementary Figure 5**: Locus Zoom plots for the five unreported novel loci

Locus Zoom plots are provided for each of the five unreported validated novel loci, showing association results from the RR-interval discovery meta-analysis in Europeans (N = 104,452). These were created using the stand-alone locus zoom plotting software (http://locuszoom.sph.umich.edu/). The linkage disequilibrium (LD) r^2^ was user-supplied and calculated in PLINK software from Exome-chip data from a subset of available studies (ASCOT-UK, ASCOT-SC, BRIGHT and 1958BC). The red line indicates a *P*-value threshold of 1x10^-5^ corresponding to the lookup significance threshold and the blue line indicates a *P*-value threshold of 2x10^-7^ corresponding to exome-wide significance.


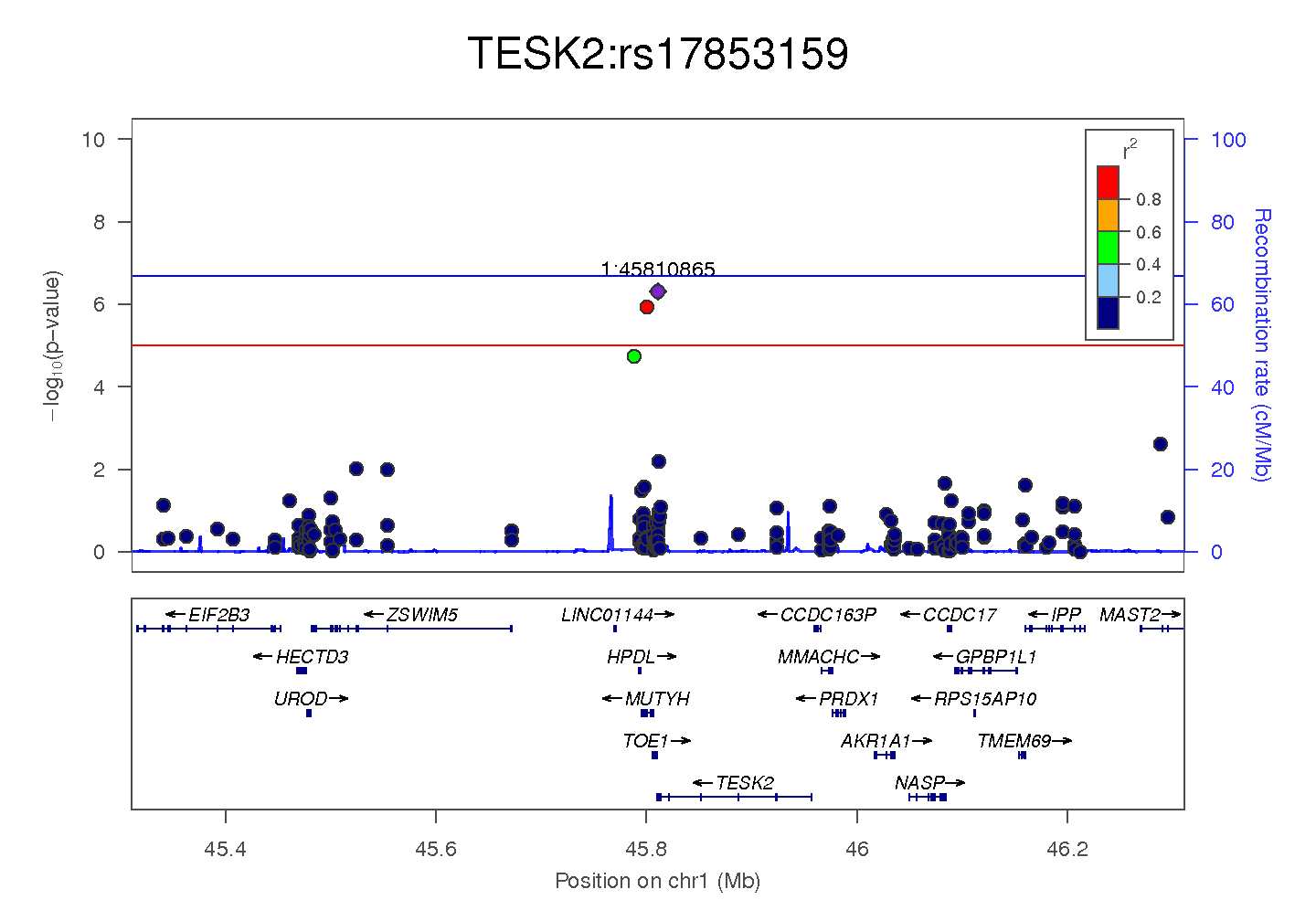

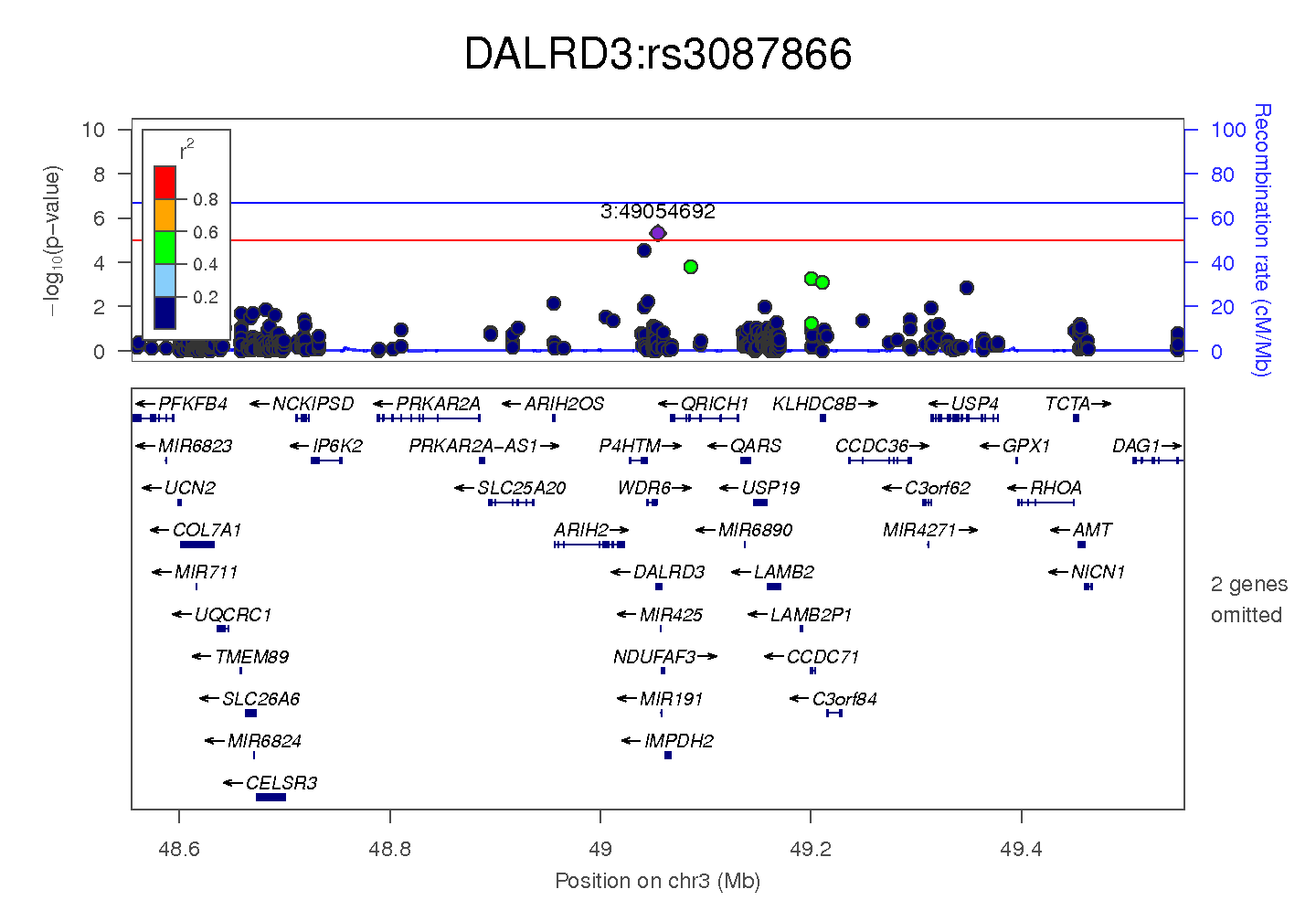


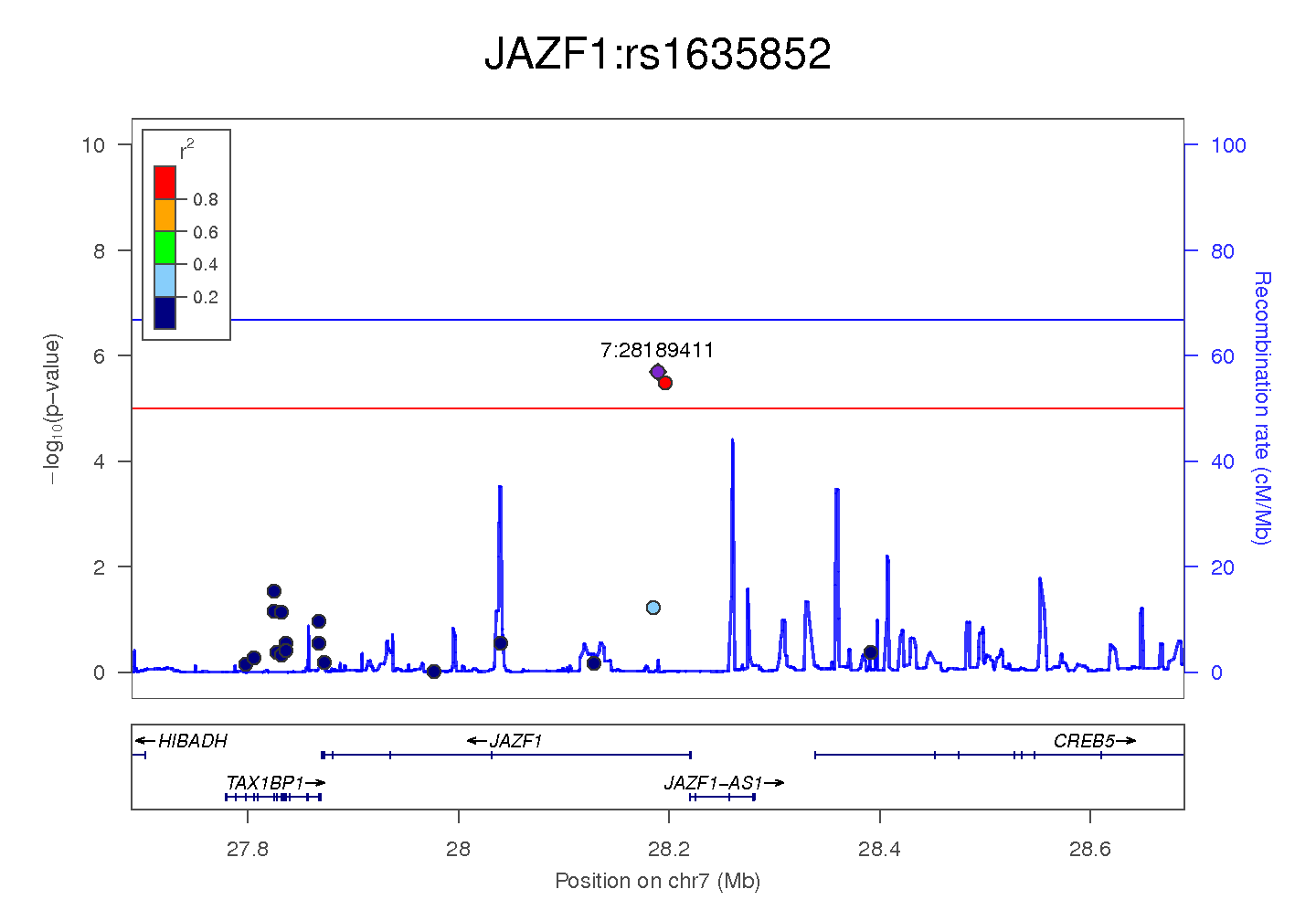

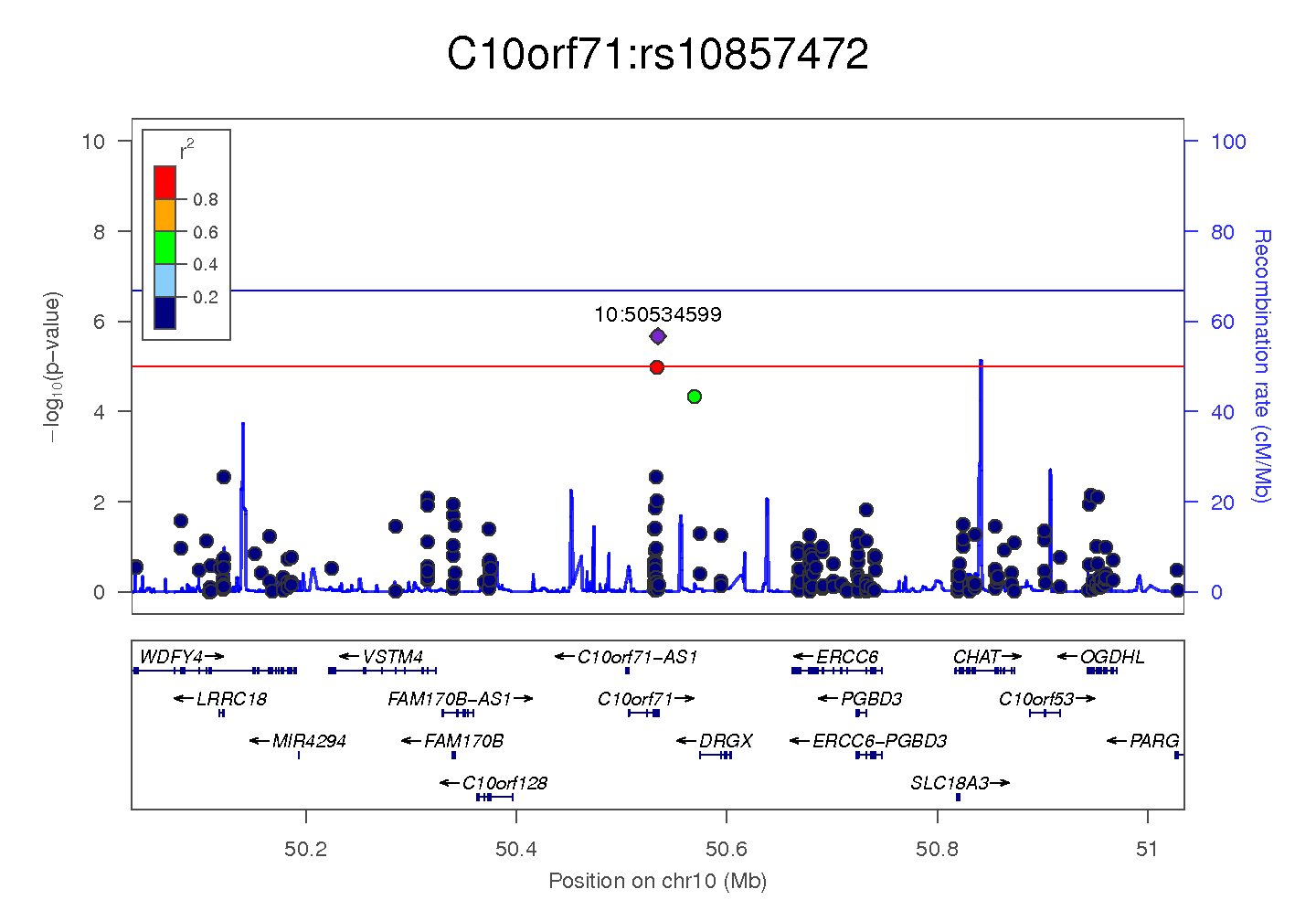


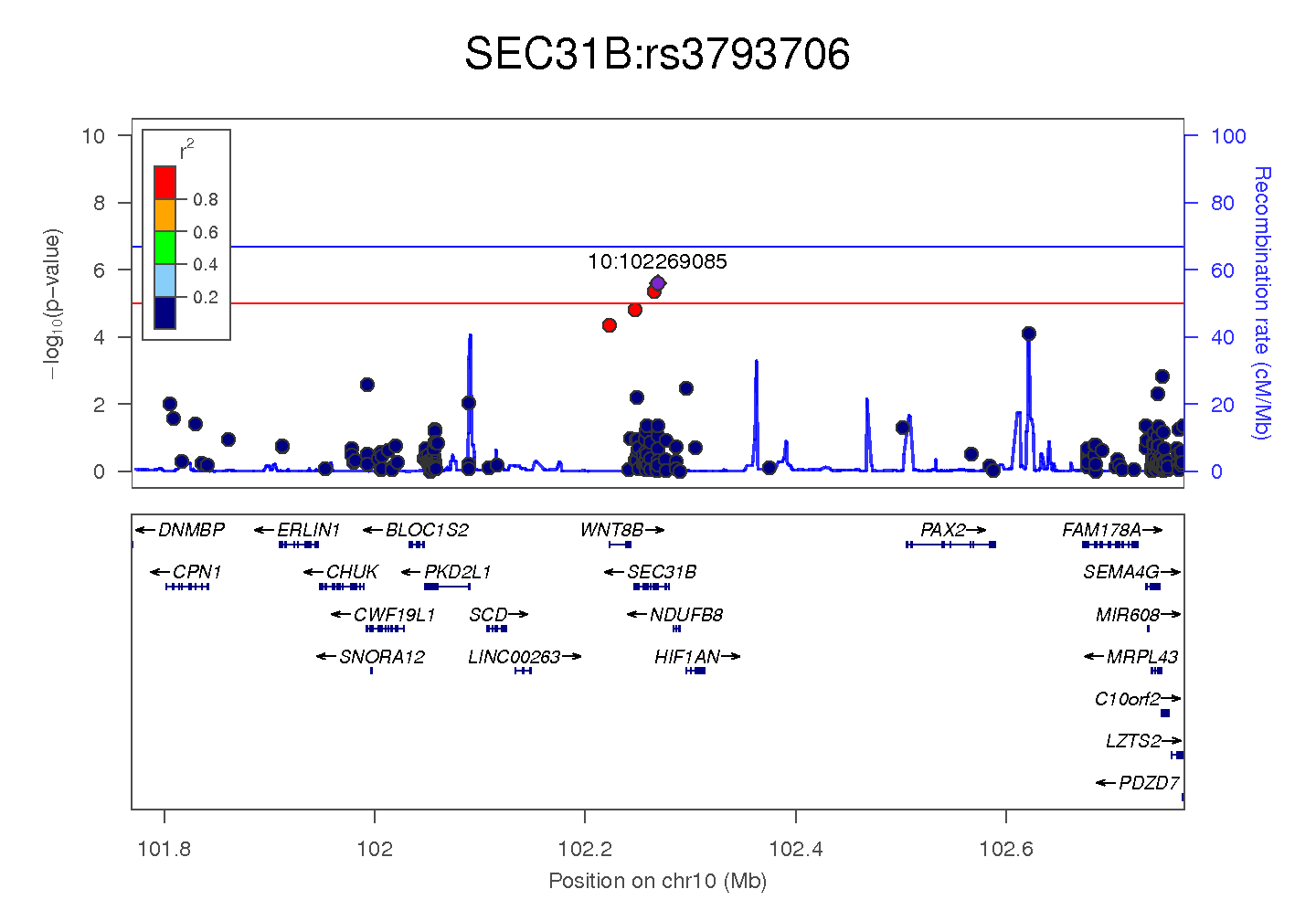


**Supplementary Figure 6**: Forest plots showing the associations of the five unreported novel loci within all European discovery cohorts and non-European studies


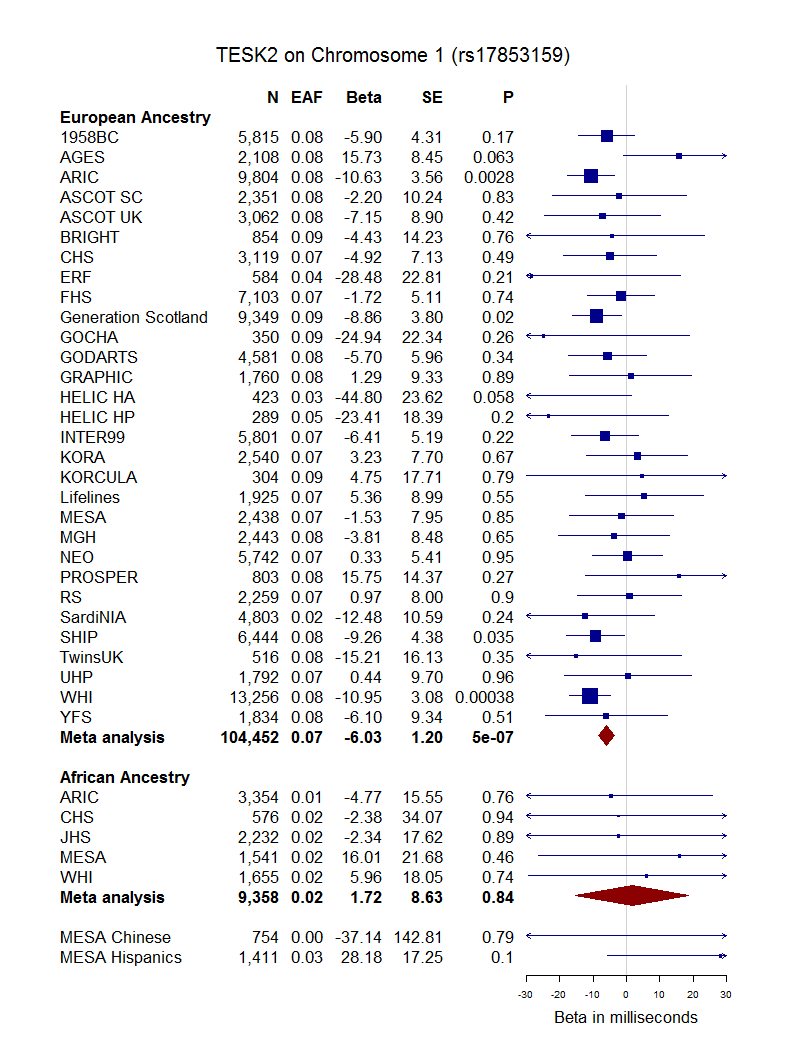

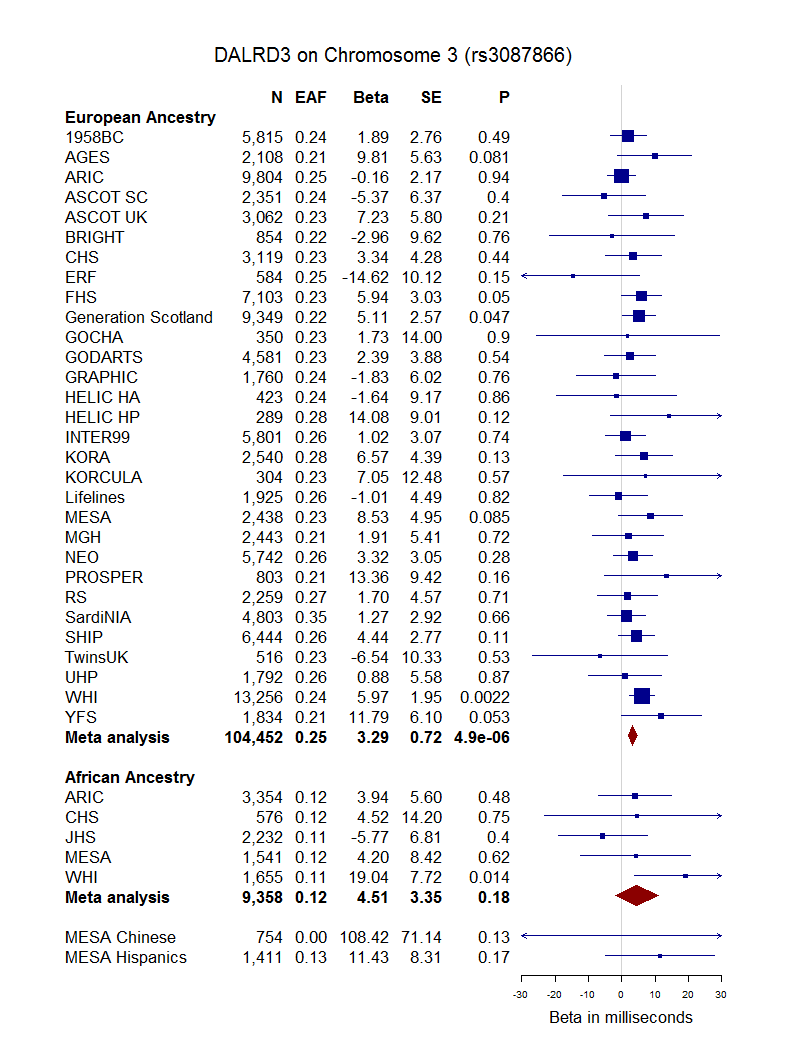

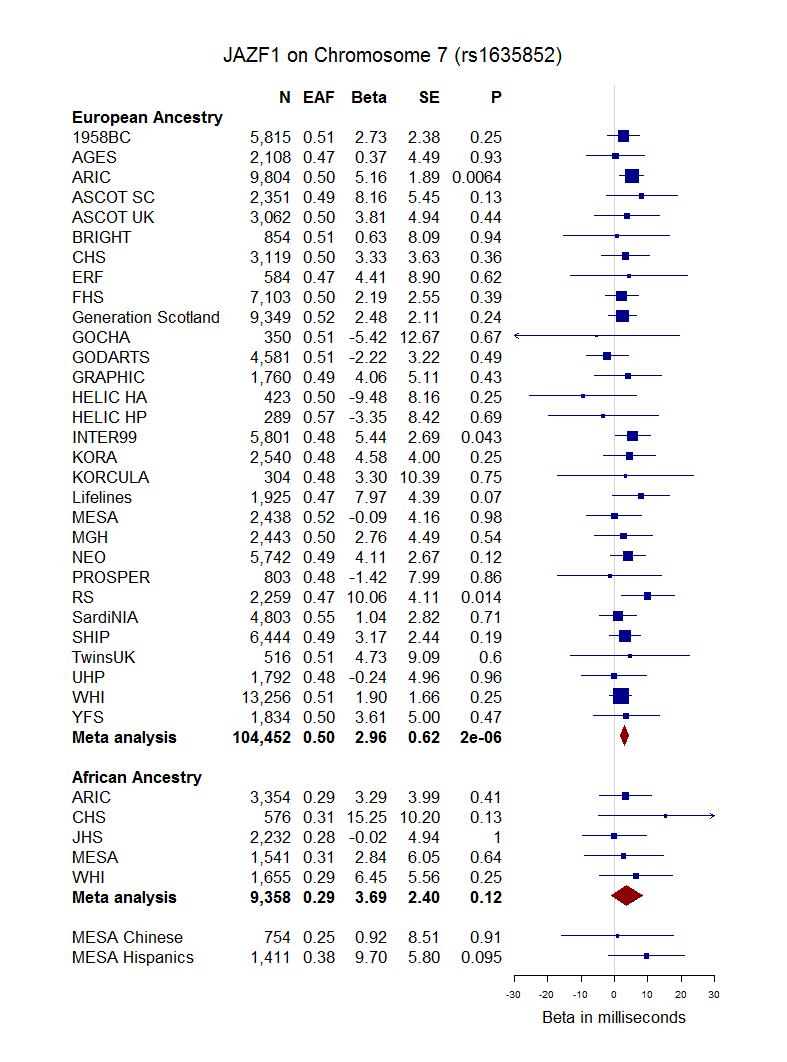

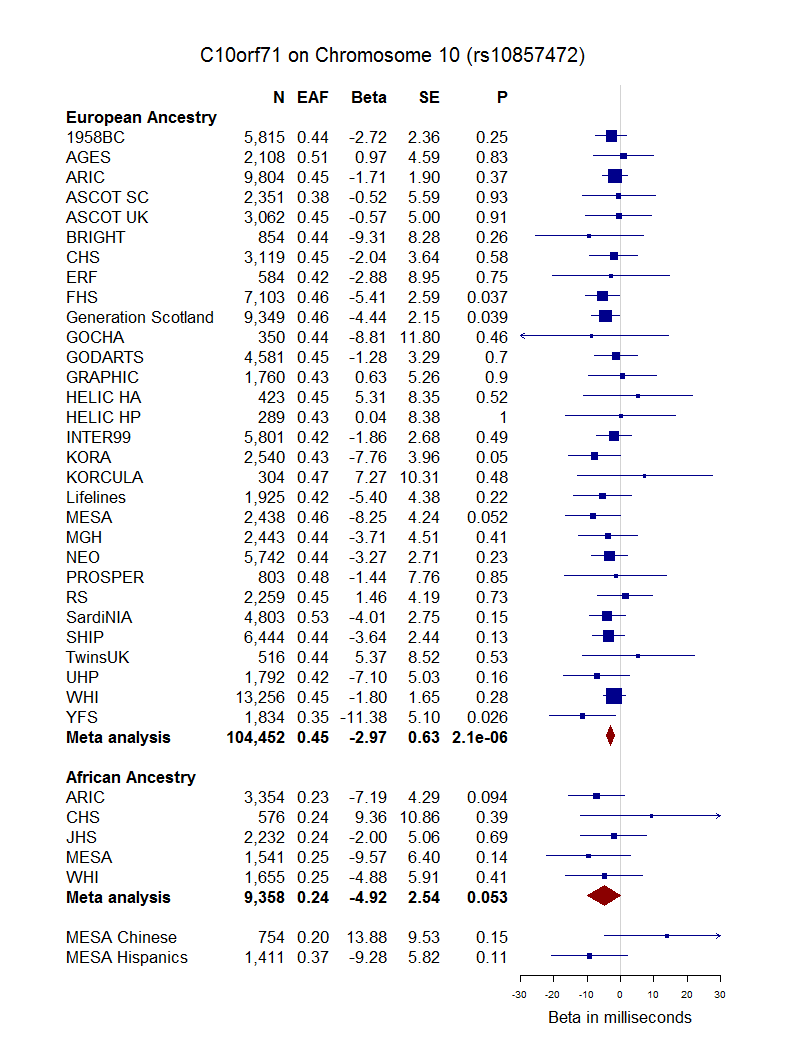

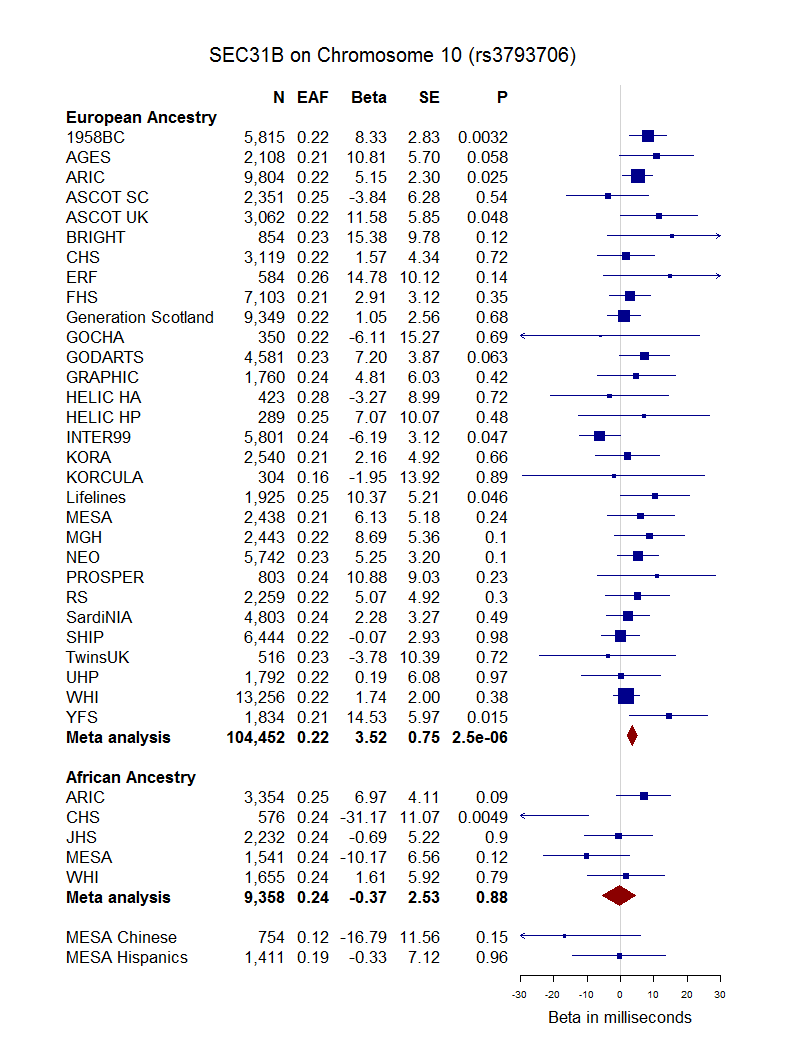


For each locus, the forest plot shows (in order): the study-level results for each of the 30 European cohorts contributing to the discovery meta-analysis; the discovery meta-analysis results; the study-level results for each of the five African ancestry cohorts; the African-ancestry meta-analysis results; the ancestry-stratified results within the MESA study for Chinese-Americans and Hispanics. In the forest plot, study-level results are displayed in blue with effect estimate boxes proportional to the sample size, and the meta-analysis results are displayed with red diamonds.

N: sample size; EAF: Effect Allele Frequency; Beta: effect size of RR-interval in milliseconds; SE: standard error; *P*: *P*-value

**Supplementary Figure 7**: Effect sizes of associated variants: comparison between nine validated novel loci and previously reported loci

**
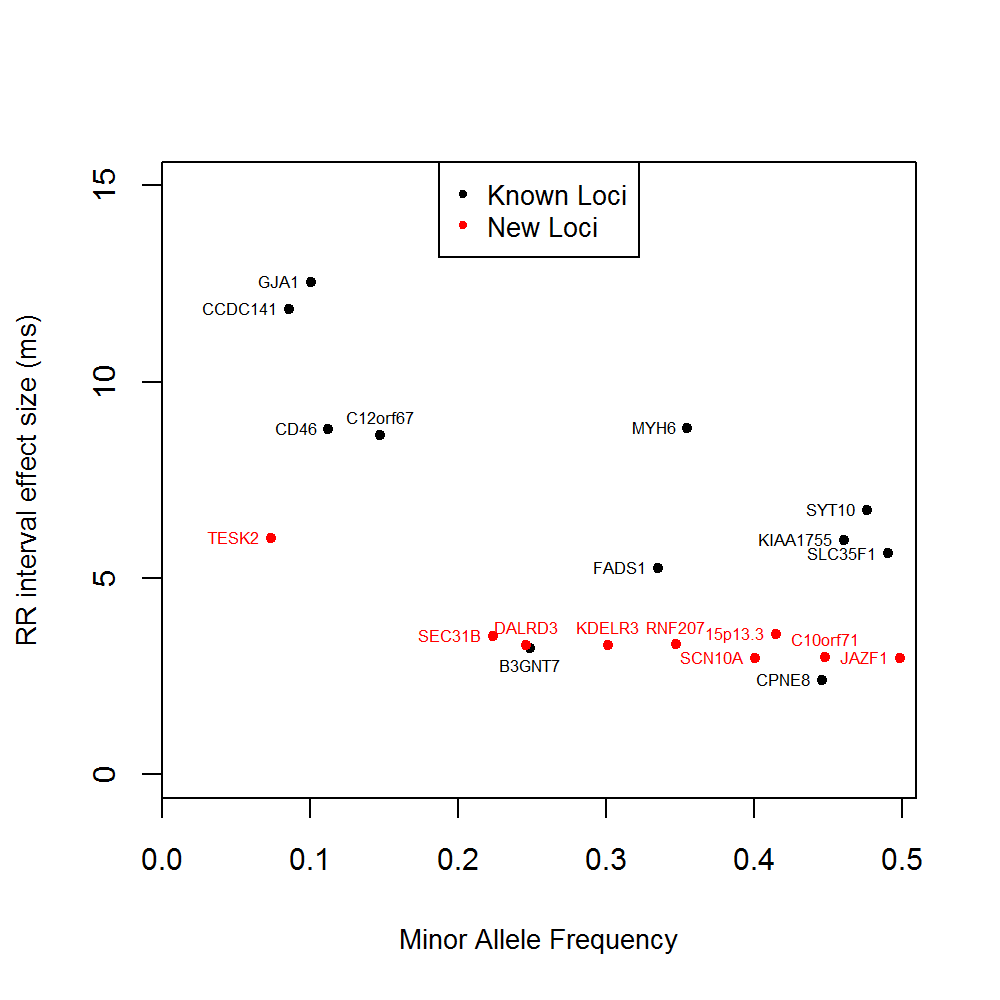
**

The effect sizes in miliseconds for association of variants with RR Interval from the primary Exome RR discovery meta-analysis in Europeans are plotted on the y-axis, with minor allele frequency (MAF) plotted on the x-axis. The variants coloured in black, denoted as “known loci”, correspond to the 11 published HR variants with strong evidence of support in our data, from the 12 previously reported GWAS variants which are covered on the Exome chip. The variants coloured in red, denoted as “new loci”, correspond to the lead variants at all nine novel loci validated in our study.
